# Supplementary material for: Allosteric regulation and crystallographic fragment screening of SARS-CoV-2 NSP15 endoribonuclease
Source: Nucleic Acids Res. 2023 Apr 28;51(10):5255–70. doi: 10.1093/nar/gkad314 (PMC10250223; doi:10.1093/nar/gkad314)
Supplement: gkad314_Supplemental_Files [file gkad314_supplemental_files.zip › Supplementary datasets.zip!/188/Refine_9/01-BUSTER/Cycle-5/LIST.html]

Refining

**BUSTER 2.X**: A suite of programs for macromolecular structure refinement by maximum-likelihood and Bayesian methods  
Copyright © 1992-2020 Global Phasing Ltd.  
All rights reserved.  
Authors: G. Bricogne, E. Blanc, M. Brandl, C. Flensburg, P. Keller, W. Paciorek, P. Roversi, O. Smart, C. Vonrhein, T. Womack  

Please reference use of the software as:

- BUSTER-TNT 2.X, Global Phasing Ltd, Sheraton House, Cambridge CB3 0AX, UK

Version: 2.10.3 for linux64-ifort made on May 20 2020 at 01:14:34  
Licenced to: Diamond Light Source, Scientific Software  

```
---


### Parsing of the BUSTER cardfile

TITLE : autoBUSTER refinement

   Cell information

   Symmetry information

 Using CCP4 X-ray form factors.

   Missing structure chemical composition

 Using ATOM block for chemical composition.


   Shell information

### Parsing of the TNT cardfile


---


### Reading observations file

 Number of reflexions in input:  47008

      Number of accepted reflexions:  40456

      Number of rejected reflexions:      0

   Preparing complete.mtz containing:      46981 reflexions


   Reflexions in test set:            2022

   Reflexions in working set:        38434


---


### 1. Generating initial model

 2.10 * Shannon sampling rate. NX NY NZ:   288  288  192


  Effective Shannon rates are:    2.44    2.44    2.23


   Allocated BUFFER of size:     61.172 Mb.

   Initial Geometry Information


 No missing atoms declared


   Summary of model volumes

 No missing atoms declared: no prior computed


 Computing bulk solvent model scattering


 NCSMASK PDB radius for solvent mask:     2.15 Angstrom

 running PDBSET (setting cell and spacegroup P1) ... done
 running NCSMASK [1] (creating mask, SMOOTH 1.14073) ... done
 running MAPDUMP (to check if reduction to unit cell is needed) ... done
 running MAPMASK (converting mask to map) ... done

   Generation of babslv by theta-filter

   Electron density in frg mask:  0.420 Å-1

 FOBS  put on rough absolute scale by Wilson plot :
           K        =  0.70832E+02
           B        =     -9.820

 

### Cycle 0001: MaxLik scaling and refinement


   MaxLik Scaling Cycle 0001

   Wilson plot

   Statistics in resolution bins just after scaling
```

|  |  |
| --- | --- |
|  | - Rxpct factors plot- Rxpct factors table- <Log-Lik Gain>- Ln Sigma A- Correlation Coefficients |

```
   <Log-Likelihood> of null-hypothesis

Structure Factor Amplitudes


   Average Figure of Merit

 Switching off refinement of overall temperature factor


 Added B =    -0.2835 Å2 to all atoms in fragment


 Added B =    -0.2835 Å2 to B_SOLV

```
gelly refinement module
 Authors (2004-2009) O. S. Smart, C. Flensburg, W. Paciorek, C. Vonrhein, 
                     M. Brandl, T. O. Womack and G. Bricogne
         (2010-2011) O. S. Smart, C. Flensburg, W. Paciorek, C. Vonrhein, 
                     T. O. Womack and G. Bricogne
         (2012-2014) O. S. Smart, C. Flensburg, W. Paciorek, C. Vonrhein, 
                     and G. Bricogne
         (2015-2020) G. Bricogne, C. Flensburg, W. Paciorek, C. Vonrhein
 Copyright © 2004-2020 Global Phasing Ltd. 
 All rights reserved.
 gelly module compilation date May 20 2020 01:11:20
 
 gelly uses routines from TNT geometry. References: 
     Tronrud, D. E., Ten Eyck, L. F., Matthews, B. W. (1987)
     "An Efficient General-Purpose Least-Squares Refinement Program 
     for Macromolecular Structures". Acta Crystallogr A, 43:489-501.
     Tronrud, D. E. (1997). "The TNT Refinement Package", 
     in Macromolecular Crystallography, Part B, Eds Charlie Carter 
     and Robert Sweet, Methods in Enzymology, 277:306-319.
 wildcard matching function by Julian Robichaux http://www.nsftools.com

 No -random_seed flag found. Random number stream initialised using:
2147483562
2147483398

 Control flags set to:  -autoncs -hydrogens_input_occ2zero \
                     -hydrogens_output_occ2heavy -screen \
                     100 -screen_sigma \
                     3.0 -keeppdb \
                     1000000 -taomonitor_verbose \
                     2 
 

 ############### Starting setup via TNT routines

 Setting up tnt routines including license check:

                   TNT Stereochemistry Program
 Version: 5.22.3.0
 Licence for Diamond Light Source, Scientific Software is valid
 
 Using TNT geometry routines to load the tnt control file: gelly.tnt
 Using TNT routines to load initial coordinates from file fragment.cor
 Expecting 6519 atoms

 Found     2194 "NOTE BUSTER_RESIDUE_ATOM_DATA" cards
 Have copied information for    6519 atoms from TNT to gelly's store 
 
 The TNT WEIGHT cards used by this run:
 -----WEIGHT       BOND     2.0
 -----WEIGHT       ANGLE    2.0
 -----WEIGHT       IMPROPER 0.0
 -----WEIGHT       TORSION  2.0
 -----WEIGHT       PSEUDO   0.0
 -----WEIGHT       TRIGONAL 2.0
 -----WEIGHT       PLANE    5.0
 -----WEIGHT       CONTACT  5.0
 -----WEIGHT       BCORREL  10.0
 -----WEIGHT       CHIRAL   5.0
 -----WEIGHT       NCS      50.0
 -----WEIGHT       IDEAL    4.0
 -----WEIGHT       RFACTOR  9.25
 
 Dictionary versions picked up from NOTE BUSTER_RESTRAINT_DICT cards as: 
 ------ protgeo_eh99.dat (V1.6.4.17) 20191001  STANDARD AMINO ACID DICTIONARY. BONDS AND ANGLES FROM ENGH AND HUBER EH99. OTHER VALUES BASED ON PREVIOUS TNT OR TAKEN FROM CCP4. INCLUDES HYDROGEN ATOMS.
 ------ exoticaa.dat (V1.3.4.15) 20200327  COLLECTION OF NON-STANDARD AMINO ACIDS, MAINLY EH91 WITHOUT IDEAL DISTANCE INFO
 ------ nuclgeo.dat (V1.13.4.8) 20190909
 ------ bcorrel.dat (V1.15.10.2) 20190616
 ------ contact.dat (V1.15.12.10) 20191121
 ------ idealdist_contact.dat (V1.3.4.12) 20191001  IDEAL-DISTANCE CONTACT TERM DATA AS USED IN PROLSQ. VALUES USED HERE ARE BASED ON DATA HARVESTING OF PDB AS OF 20190109.
 ------ restraints for LIG (Unknown) from cif dictionary Z1262327505.cif
 ------ restraints for CIT (CITRIC ACID) from cif dictionary CIT.cif; generated by GRADE_PDB_LIGAND 1.2.12 (October 4 2016) using MOGUL 1.7.2(RC1), CSD as538be, with quantum mechanics RM1 ((DatabaseID PDB-CIT))
 ------ assume.dat (V1.9.4.2) 20180705
 
 Have picked up spacegroup name for pdb output as 'P 63      '
 Setting up Chain/Residue arrays
 
 Have command line specification -autoncs*
     Have found       1 NCS relations
     Pairwise matching residue number and type between chains: 
                    A        B        S        W    
          A         .      100%       -        -   
          B                  .        -        -   
          S                           .        -   
          W                                    .   
       Table key--                                                       
          all   : two chains have 100% match and same number of residues 
         multi  : ditto and they contribute to a multi chain ncs cluster 
          ***%  : two chains have >80% matching residue number/type      
           -    : chains appear to be unrelated                          
      chain A     have identified ncs to     1 other chains. So will weight each autoncs by (       6.666/#)=      6.6660
      chain B     have identified ncs to     1 other chains. So will weight each autoncs by (       6.666/#)=      6.6660
      chain S     have identified ncs to     0 other chains. 
      chain W     have identified ncs to     0 other chains. 
     For .Gelly cards produced see below - after pruning
 
 Setting up BUSTER_SET groups
 Have found    89 BUSTER_SET cards
 Now silently applying automatic/dictionary set definitions. For a verbose listing use '-verbose_set' flag.
 Applying user defined set definitions:
 =====NOTE BUSTER_SET ncsautoXcld = Water --> set 'ncsautoXcld' now has   966 atoms 
 =====NOTE BUSTER_SET ncsautoABset = Chain_A \ ncsautoXcld --> set 'ncsautoABset' now has  2783 atoms 
 Have defined    49 sets: 
      set:                 'All' has   6519 atoms in   1667 residues - automatically defined
      set:               'Empty' has      0 atoms in      0 residues - automatically defined
      set:            'ExoticAA' has      0 atoms in      0 residues - defined by dictionary file
      set:           'ExoticNUC' has      0 atoms in      0 residues - defined by dictionary file
      set:              'NotHET' has   5514 atoms in    698 residues - defined by dictionary file
      set:             'Chain_A' has   2783 atoms in    351 residues - automatically defined
      set:             'Chain_B' has   2770 atoms in    350 residues - automatically defined
      set:             'Chain_S' has    567 atoms in    567 residues - automatically defined
      set:             'Chain_W' has    399 atoms in    399 residues - automatically defined
      set:               'Water' has    966 atoms in    966 residues - defined by dictionary file
      set:          'StdProtein' has   5514 atoms in    698 residues - defined by dictionary file
      set:             'Protein' has   5514 atoms in    698 residues - defined by dictionary file
      set:                'Back' has   2794 atoms in    698 residues - defined by dictionary file
      set:                'Side' has   2720 atoms in    650 residues - defined by dictionary file
      set:        'RnaCanonical' has      0 atoms in      0 residues - defined by dictionary file
      set:        'DnaCanonical' has      0 atoms in      0 residues - defined by dictionary file
      set:     'RnaDnaCanonical' has      0 atoms in      0 residues - defined by dictionary file
      set:     'DnaRnaCanonical' has      0 atoms in      0 residues - defined by dictionary file
      set:         'RnaExtended' has      0 atoms in      0 residues - defined by dictionary file
      set:         'DnaExtended' has      0 atoms in      0 residues - defined by dictionary file
      set:      'RnaDnaExtended' has      0 atoms in      0 residues - defined by dictionary file
      set:      'DnaRnaExtended' has      0 atoms in      0 residues - defined by dictionary file
      set:                 'Dna' has      0 atoms in      0 residues - defined by dictionary file
      set:                 'Rna' has      0 atoms in      0 residues - defined by dictionary file
      set:              'RnaDna' has      0 atoms in      0 residues - defined by dictionary file
      set:              'DnaRna' has      0 atoms in      0 residues - defined by dictionary file
      set:             'Nucleic' has      0 atoms in      0 residues - defined by dictionary file
      set:             'Polymer' has   5514 atoms in    698 residues - defined by dictionary file
      set: 'BufferWeichenberger' has     26 atoms in      2 residues - defined by dictionary file
      set:    'BufferAdditional' has      0 atoms in      0 residues - defined by dictionary file
      set:              'Buffer' has     26 atoms in      2 residues - defined by dictionary file
      set:         'MetalAlkali' has      0 atoms in      0 residues - defined by dictionary file
      set:  'MetalAlkalineEarth' has      0 atoms in      0 residues - defined by dictionary file
      set:     'MetalTransition' has      0 atoms in      0 residues - defined by dictionary file
      set: 'MetalPostTransition' has      0 atoms in      0 residues - defined by dictionary file
      set:    'MetalLanthanides' has      0 atoms in      0 residues - defined by dictionary file
      set:               'Metal' has      0 atoms in      0 residues - defined by dictionary file
      set:         'SugarAlphaD' has      0 atoms in      0 residues - defined by dictionary file
      set:         'SugarAlphaL' has      0 atoms in      0 residues - defined by dictionary file
      set:               'Sugar' has      0 atoms in      0 residues - defined by dictionary file
      set:               'Other' has     39 atoms in      3 residues - defined by dictionary file
      set:            'Hydrogen' has      0 atoms in      0 residues - defined by dictionary file
      set:         'nonHydrogen' has   6519 atoms in   1667 residues - defined by dictionary file
      set:             'OccZero' has      0 atoms in      0 residues - defined by dictionary file
      set:          'OccNonZero' has   6519 atoms in   1667 residues - defined by dictionary file
      set:             'OccFull' has   6474 atoms in   1664 residues - defined by dictionary file
      set:          'OccNonFull' has     45 atoms in      3 residues - defined by dictionary file
      set:         'ncsautoXcld' has    966 atoms in    966 residues - user specified
      set:        'ncsautoABset' has   2783 atoms in    351 residues - user specified
 No command line arguments '-occ_allhet' or '-occ_restyp' so will not setup automated occcupancy refinement
 
 Interpreting CONSTANT and FREE cards: 
 =====CONSTANT     OCC
 Have found and interpreted      1 CONSTANT cards in the TNT input. 
 Before any COMBINE - Number of atoms with fixed XYZ =     0
 Before any COMBINE - Number of atoms with fixed B   =     0
 Before any COMBINE - Number of atoms with fixed OCC =  6519
 Before any COMBINE - Number of atoms with fixed BIJ =   966
                            cf total number of atoms =  6519
 
 There are no COMBINE cards to be found in the TNT card input 
 
 There are no NOTE BUSTER_COMBINE_RES cards to be found in the TNT card input 

 ############### Working out full connectivity list


 ############### Working out equivalent atoms


 Residue with most atoms given below:
        A|58:N(TRP)         
        A|58:CA(TRP)        
        A|58:CB(TRP)        
        A|58:CG(TRP)        
        A|58:CD1(TRP)       
        A|58:NE1(TRP)       
        A|58:CE2(TRP)       
        A|58:CD2(TRP)       
        A|58:CE3(TRP)       
        A|58:CZ3(TRP)       
        A|58:CH2(TRP)       
        A|58:CZ2(TRP)       
        A|58:C(TRP)         
        A|58:O(TRP)         


 Have no '-equiv_check_plus' argument - so will take atom type into
 account when checking for equivalence of otherwise identical atoms.
 

 ############### Looking at dihedrals


 ############### Looking at chirals

 NOTE BUSTER_CHIRAL_ANGLECUT card found - read value=    -31.00 degs from it
 NOTE BUSTER_CHIRAL_SIGMA card found - read value=      1.70 degs from it
 RANGE allowed for B variables in refinement:   from      3.00000 to    300.00000

 ############### Preparing for PDB output


 ############### Processing extra restraints explicitly defined

 No 'NOTE BUSTER_TARGET' cards or -target?? args found (used to read coordinates files for soft ncs to external)
 No 'NOTE BUSTER_OCCSUM'     cards found - so not using sum of occupancy restraints
 No 'NOTE BUSTER_DISTANCE'   cards found - so not setting up any additional atom-atom distance restraints
 No 'NOTE BUSTER_UTILANGLE'  cards found - so not setting up any additional restraints on any angle
 No 'NOTE BUSTER_UTILTOR'    cards found - so not setting up any additional sinusoidal restraints on torsions
 
 --- Have some similarity definitions 
     Similarity restraints on RMSD use superposition routines from: 
     Coutsias, E.A., Seok, C., Dill, K.A.(2004)
     "Using quaternions to calculate RMSD", J. Comput. Chem., 25:1849-1857 

 Have found    1 'NOTE BUSTER_SIM_DEFINE' cards 
     Maximum number of chains involved in any SIM group (excluding the template)=     1


 ########## Interpreting card: NOTE BUSTER_SIM_DEFINE ncsautoAB ncsautoABset B
     Number of atoms in template set=  2783
     Number of related chains (other than template) in SIM set=    1
     List of chains to be restrained (excluding template)=   B   
     Pairwise RMSD, XYZ in Angstroms, for SIM group: ncsautoAB
                 A         B   
     A         -----     ----- 
     B         0.699     ----- 
     Overall RMSD,XYZ for the SIM group is     0.699 Angstroms
     Pairwise rms deviation of [individual B's minus their set/chain average] in Angs**2
                 A         B         Bavg
     A         -----     -----    102.82
     B         33.06     -----     78.60
     Overall RMSD,B   for the SIM group is    33.059 Angs**2
     The number of common atoms for the above fits:
                 A         B   
     A         -----     ----- 
     B          2770     ----- 
     (compared to the template set that has    2783 atoms in it)
     List of atoms that are absent in one or more chains for this group: 
                                    A    B   
                    C01     501     +    -
                    N02     501     +    -
                    C03     501     +    -
                    C04     501     +    -
                    C05     501     +    -
                    C06     501     +    -
                    C07     501     +    -
                    N08     501     +    -
                    C09     501     +    -
                    C10     501     +    -
                    C11     501     +    -
                    N12     501     +    -
                    C13     501     +    -
     (key  +  atom present for this chain 
           -  atom absent  for this chain)
 transforms for each of the superpositions orthogonal matrix r11 r12 r13 r21 r22 r23 r31 r32 r33 tx ty tz (PDBSET syntax): 
TRANS_AB -0.873874 -0.485259 -0.029479 -0.485290  0.874329 -0.006578  0.028967  0.008557 -0.999544    33.967    33.023    14.835
 reverse transforms 
TRANS_BA -0.873874 -0.485290  0.028967 -0.485259  0.874329  0.008557 -0.029479 -0.006578 -0.999544    45.279   -12.517    16.047
 
 Setting up LSSR interatomic distance restraints
 Interpreting card: NOTE BUSTER_SIM_RESTRAIN_LSSR ncsautoAB 6.666
        setting up interatomic distance pair restraints with:
        Dmin=         0.00
        Dmax=         5.50
        sigma=        0.20
        Vmax=         3.00
        weight=       6.67
        number of distance pairs defined for this set is      35151
 Have not supplied either '-sim_swap_equiv' or '-sim_swap_equiv_plus' argument 
   so will not try swaping equivalent atoms to improve NCS and/or target agreement.
 
 NCS by LSSR outlier analysis
 -autoncs option automatic NCS prune routine -------- 
 Residues pruned out by gradient condition:
           A|54     A|131     A|135     A|140     A|148     A|149     A|151
          A|158     A|168     A|169     A|172     A|174     A|175     A|181
          A|182     A|184     A|185     A|186     A|187     A|189     A|216
          A|217     A|218     A|219     A|220     A|221     A|222     A|237
          A|250     A|253     A|255     A|256     A|259     A|260     A|264
          A|318     A|320     A|322     A|325     A|326     A|327     A|328
          A|329     A|344     A|345      B|54     B|131     B|135     B|140
          B|148     B|151     B|158     B|168     B|169     B|172     B|174
          B|175     B|181     B|182     B|184     B|185     B|186     B|187
          B|189     B|216     B|217     B|218     B|219     B|220     B|221
          B|222     B|237     B|250     B|253     B|255     B|256     B|259
          B|260     B|264     B|318     B|320     B|322     B|325     B|326
          B|327     B|328     B|329     B|344     B|345
  
 Residues pruned out by function/max condition:
          A|149!    A|168!    A|169!    A|171     A|172!    A|173     A|174!
          A|187!    A|188     A|220!    A|221!    A|259!    A|264!    A|326!
          A|346    A|ENDA     A|401     B|149     B|168!    B|169!    B|171 
          B|172!    B|173     B|174!    B|187!    B|188     B|220!    B|221!
          B|259!    B|264!    B|326!    B|346    B|ENDA     B|401 
  
 Total number of residues for which LSSR restraints have been pruned out is      102 out of      700 participating
 Number of LSSR restraints pruned out is      7450 this is   21.2% of the total           35151
  
 -autoncs .Gelly cards suitable for adaptation for manual definition/pruning
           (note auto pruning is done in a slightly different way so exact results may differ slightly)
 -autoncs option .Gelly cards also written to file  auto_gelly_cards.txt
NOTE BUSTER_SET ncsautoXcld = Water    # card added - produced by -autoncs
NOTE BUSTER_SET ncsautoXcld = ncsautoXcld + { A|54 A|131 A|135 A|140 A|148 }    # card added - produced by -autoncs
NOTE BUSTER_SET ncsautoXcld = ncsautoXcld + { A|149 A|151 A|158 A|168 A|169 }    # card added - produced by -autoncs
NOTE BUSTER_SET ncsautoXcld = ncsautoXcld + { A|171 A|172 A|173 A|174 A|175 }    # card added - produced by -autoncs
NOTE BUSTER_SET ncsautoXcld = ncsautoXcld + { A|181 A|182 A|184 A|185 A|186 }    # card added - produced by -autoncs
NOTE BUSTER_SET ncsautoXcld = ncsautoXcld + { A|187 A|188 A|189 A|216 A|217 }    # card added - produced by -autoncs
NOTE BUSTER_SET ncsautoXcld = ncsautoXcld + { A|218 A|219 A|220 A|221 A|222 }    # card added - produced by -autoncs
NOTE BUSTER_SET ncsautoXcld = ncsautoXcld + { A|237 A|250 A|253 A|255 A|256 }    # card added - produced by -autoncs
NOTE BUSTER_SET ncsautoXcld = ncsautoXcld + { A|259 A|260 A|264 A|318 A|320 }    # card added - produced by -autoncs
NOTE BUSTER_SET ncsautoXcld = ncsautoXcld + { A|322 A|325 A|326 A|327 A|328 }    # card added - produced by -autoncs
NOTE BUSTER_SET ncsautoXcld = ncsautoXcld + { A|329 A|344 A|345 A|346 A|ENDA }    # card added - produced by -autoncs
NOTE BUSTER_SET ncsautoXcld = ncsautoXcld + { A|401 }    # card added - produced by -autoncs
NOTE BUSTER_SET ncsautoABset = Chain_A \ ncsautoXcld    # card added - produced by -autoncs
NOTE BUSTER_SIM_DEFINE ncsautoAB ncsautoABset B    # card added - produced by -autoncs
NOTE BUSTER_SIM_RESTRAIN_LSSR ncsautoAB 6.666    # card added - produced by -autoncs

 LSSR function contribution before pruning     61180.2
                             after pruning      7373.1
 Graph of which residues pruned from LSSR: ncs_pruned.mtv
 Graph of LSSR function outliers ncs_function_outliers.mtv
 Graph of LSSR gradient outliers ncs_gradient_outliers.mtv
 LSSR - analysis of distance difference distribution for NCS
         DeltaD less than 0.5 Angs:  proportion of population= 99.19% average DeltaD of subset     0.014 Angs
 Histogram of LSSR deltaD distribution ncs_histogram.mtv
 
 Interpreting card: NOTE BUSTER_TLS_SET tls1 { A|* }
 Interpreting card: NOTE BUSTER_TLS_SET tls2 { B|* }
 Interpreting card: NOTE BUSTER_TLS_SET tls1 { A|* }
 Interpreting card: NOTE BUSTER_TLS_T   tls1     -2.44720E-01 -1.33885E-01  3.49464E-01 -7.26320E-02  1.36549E-02 -3.31073E-02
 Interpreting card: NOTE BUSTER_TLS_L   tls1      2.21033E+00  2.43686E+00  1.90206E+00 -4.70482E-01 -1.42181E+00  6.26775E-01
 Interpreting card: NOTE BUSTER_TLS_S   tls1      1.26526E-01 -3.75851E-01  4.70780E-01 -3.29027E-01  1.72095E-01 -3.71904E-01  6.80978E-02 -3.47299E-01
 Interpreting card: NOTE BUSTER_TLS_O   tls1     -2.31731E+01 -8.40215E+01  1.10770E+01
 Interpreting card: NOTE BUSTER_TLS_FIX tls1 ALL
 Interpreting card: NOTE BUSTER_TLS_SET tls2 { B|* }
 Interpreting card: NOTE BUSTER_TLS_T   tls2     -3.51018E-01 -2.65873E-01  4.05020E-01  2.48330E-02  3.11379E-02 -4.04722E-02
 Interpreting card: NOTE BUSTER_TLS_L   tls2      1.80307E+00  1.69393E+00  1.39140E+00  8.83117E-01 -1.11732E+00 -9.56109E-01
 Interpreting card: NOTE BUSTER_TLS_S   tls2     -1.42162E-02  9.70000E-02 -1.62700E-01  8.39808E-02  2.10687E-01  5.67053E-02 -9.55320E-02  7.37692E-02
 Interpreting card: NOTE BUSTER_TLS_O   tls2      2.22040E+01 -7.22942E+01  3.58164E+00
 Interpreting card: NOTE BUSTER_TLS_FIX tls2 ALL


 TLS information. Total number of groups:     2


 Group   Natoms    FixRB   FixTLS   Group Tag    Group Spec
     1     2783      Y        Y     tls1    { A|* }
     2     2770      Y        Y     tls2    { B|* }
 
NOTE BUSTER_TLS_SET tls1 { A|* }
NOTE BUSTER_TLS_T   tls1     -2.44720E-01 -1.33885E-01  3.49464E-01 -7.26320E-02  1.36549E-02 -3.31073E-02
NOTE BUSTER_TLS_L   tls1      2.21033E+00  2.43686E+00  1.90206E+00 -4.70482E-01 -1.42181E+00  6.26775E-01
NOTE BUSTER_TLS_S   tls1      1.26526E-01 -3.75851E-01  4.70780E-01 -3.29027E-01  1.72095E-01 -3.71904E-01  6.80978E-02 -3.47299E-01
NOTE BUSTER_TLS_O   tls1     -2.31731E+01 -8.40215E+01  1.10770E+01
NOTE BUSTER_TLS_FIX tls1 ALL
 
NOTE BUSTER_TLS_SET tls2 { B|* }
NOTE BUSTER_TLS_T   tls2     -3.51018E-01 -2.65873E-01  4.05020E-01  2.48330E-02  3.11379E-02 -4.04722E-02
NOTE BUSTER_TLS_L   tls2      1.80307E+00  1.69393E+00  1.39140E+00  8.83117E-01 -1.11732E+00 -9.56109E-01
NOTE BUSTER_TLS_S   tls2     -1.42162E-02  9.70000E-02 -1.62700E-01  8.39808E-02  2.10687E-01  5.67053E-02 -9.55320E-02  7.37692E-02
NOTE BUSTER_TLS_O   tls2      2.22040E+01 -7.22942E+01  3.58164E+00
NOTE BUSTER_TLS_FIX tls2 ALL


 TLS: mmCIF group specification begin


#
loop_
_pdbx_refine_tls_group.id
_pdbx_refine_tls_group.refine_tls_id
_pdbx_refine_tls_group.beg_PDB_ins_code
_pdbx_refine_tls_group.beg_auth_asym_id
_pdbx_refine_tls_group.beg_auth_seq_id
_pdbx_refine_tls_group.end_PDB_ins_code
_pdbx_refine_tls_group.end_auth_asym_id
_pdbx_refine_tls_group.end_auth_seq_id
_pdbx_refine_tls_group.selection
_pdbx_refine_tls_group.selection_details
   1   1 . A   -1 . A  346 . '{ A|* }'
   2   1 . A  401 . A  501 . '{ A|* }'
   3   2 . B   -1 . B  346 . '{ B|* }'
   4   2 . B  401 . B  401 . '{ B|* }'
#


 TLS: mmCIF group specification end


 Interpreting card: NOTE BUSTER_ANISO_SET Empty
 Interpreting card: NOTE BUSTER_ANISO_WEIGHT SCA  16.0
 Interpreting card: NOTE BUSTER_ANISO_WEIGHT SPH   0.6    0.6
 Interpreting card: NOTE BUSTER_ANISO_WEIGHT SIM   7.8    4.8    2.6    0
 Interpreting card: NOTE BUSTER_ANISO_WEIGHT RIG  21.5   21.5    3.7    0


 After TLS and ANISO: Number of atoms with fixed/combined xyz =     0
 After TLS and ANISO: Number of atoms with fixed/combined B   =     0
 After TLS and ANISO: Number of atoms with fixed/combined OCC =  6519
 After TLS and ANISO: Number of atoms with fixed/combined BIJ =  6519
 After TLS and ANISO: Number of atoms in a TLS group          =  5553
                                     cf total number of atoms =  6519
 

 ############### Setup state vector

 No free_vect_ulim_xyz argument found: using default of  100.000
 No free_vect_llim_xyz argument found: using default of -100.000
 Number of parameters added for TLS description                 =       0
 Number of parameters to describe molecular conformation        =   26076

 ############### Setup free vector

 Number of free parameters to be adjusted in refinement   =   26076

 ############### Setting up own geometry


 No command line argument '-tnt_fn' so will use gelly to do geometry function calculations

 ============= Call TNT functions


 ============= Get TNT symmetry information

 
      Getting symmetry operators from TNT. 
          gelly will classify symmetry using pdb-like convention: 
               SYMOP   SYMMETRY
              NNNMMM   OPERATOR
                1555   X,Y,Z
                2555   -Y,X-Y,Z
                3555   -X+Y,-X,Z
                4555   -X,-Y,1/2+Z
                5555   Y,-X+Y,1/2+Z
                6555   X-Y,X,1/2+Z
          where NNN -> operator number and MMM -> translation vector
 

 ============= Own Gelly geometry/restraints setup


 ----------- Identify bond-types


    Setting up GEOMBONDREC_TYPE_HASH* to be able to map a given bond type (e.g.
    "single") to an internal integer (GBOND_TYPE=1) etc.

    Using default  GEOMBONDREC_TYPE_HASH_STR='SING* 1 DOUB* 2 TRIP* 3 AROM* 4 DELO* 4 METAL* 5':
    to override default use a NOTE BUSTER_GEOMBONDREC_TYPE_HASH_STR gelly card.

 ----------- Classify torsion into peptide omega and other


 ----------- Setup of non-bonded terms

 Ideal-distance contact term setup:
    Restraint function constants (normally these values are from NOTE BUSTER_IDEAL_CONTACT_* cards)
    Sigma for general contacts      0.200 Angs
    Sigma for 1-4 contacts          0.100 Angs (contacts across a torsion)
    Sigma for 1-5 contacts          0.150 Angs
    Sigma for 1-N contacts          0.200 Angs
    Sigma for HBOND pairs           0.100 Angs (contacts between HB_types A and D or B's)
    Contact distance increment for each atom in HBOND          -0.150 Angs 
    Contact distance increment for H-bond acceptor              0.100 Angs 
    Contact distance increment for 1-4 with an O atom          -0.050 Angs 
    Contact distance increment for 1-4 with an N atom          -0.180 Angs 
    Contact distance increment for 1-4 with an C atom          -0.370 Angs 
    Contact distance increment for 1-4 with an S atom          -0.150 Angs 
    Contact distance increment for 1-4 with an P atom          -0.150 Angs 
    Contact distance increment for 1-4 with any other atom     -0.150 Angs 
    All atoms matched for CCP4 atom type.
    Number of CCP4 atom types used is         23 (H-bound and not H-bound counted separately)

 Setting up short/ideal-distance contacts
   Using gelly special position handling. (For TNT instead rerun with argument '-special_tnt')

   No command line argument '-special_all' specified. We will auto-generate
   EXCLUDE cards only for atom-atom contacts across symmetry (i.e. on special
   position) when this involves a contact within a residue to itself.

   Have set up non-bonded exclusions - 1-3 contacts
   Atom A|27:CA         makes the most 1-3 contacts:  6

   Have set up non-bonded exclusions - 1-4 contacts
   Atom A|27:N          makes the most 1-4 contacts:  7

   Have set up non-bonded exclusions - 1-5 contacts
   Atom A|28:C.A        makes the most 1-5 contacts: 10

 ############################################################################################################
 ###################################################### setting up contact terms
 ############################################################################################################


 ============= Call Gelly versions of TNT functions

 WARNING: 1-4 contact without 1-(2,3)-4 data for A|401:C2(CIT)        and A|401:O6(CIT)        X-X
 WARNING: 1-4 contact without 1-(2,3)-4 data for A|401:O7(CIT)        and A|401:C5(CIT)        X-X
 WARNING: 1-4 contact without 1-(2,3)-4 data for A|401:O7(CIT)        and A|401:O5(CIT)        X-X
 WARNING: 1-4 contact without 1-(2,3)-4 data for A|401:C4(CIT)        and A|401:O6(CIT)        X-X
 WARNING: 1-4 contact without 1-(2,3)-4 data for A|501:C01(LIG)       and A|501:N08(LIG)       X-X
 WARNING: 1-4 contact without 1-(2,3)-4 data for A|501:C01(LIG)       and A|501:C11(LIG)       X-X
 WARNING: 1-4 contact without 1-(2,3)-4 data for A|501:N02(LIG)       and A|501:C10(LIG)       X-X
 WARNING: 1-4 contact without 1-(2,3)-4 data for B|401:C1(CIT)        and B|401:C4(CIT)        X-X
 WARNING: 1-4 contact without 1-(2,3)-4 data for B|401:C2(CIT)        and B|401:O6(CIT)        X-X
 WARNING: 1-4 contact without 1-(2,3)-4 data for B|401:O7(CIT)        and B|401:O5(CIT)        X-X
 WARNING: 1-4 contact without 1-(2,3)-4 data for B|401:C4(CIT)        and B|401:O6(CIT)        X-X

       TERM      weight      number      TNT_fn    Gelly_fn        diff
       BOND       2.000        5668    1994.379    1994.379       0.000
       ANGL       2.000        7691    6017.315    6017.315       0.000
       IMPR       0.000           0       0.000       0.000       0.000
       TORS       2.000        1973    3672.518    3672.518      -0.000
       TRIG       2.000           0       0.000       0.000       0.000
       PLAN       5.000         967    2399.352    2399.352       0.000
       CONT       5.000           0     144.048       0.000     144.048
       BCOR      10.000        5630   10983.008    9483.528    1499.480


 ============= Call Gelly geometry functions

   Switching torsion functional form to sinusoidal rather than split harmonic functional.
      if you want this not to happen then rerun with '-torsharm'
      Weight for sinusoidal torsion=     2.000
   BCOR function/gradient will be split between H and non-H atoms (as for any atom pair). Use '-bcorrel_all_in_hydrogens' to change this.

 ----------- Fix B-correlation restraints

 
 Checking for bonds that do not have a bcorrel restraint defined
 We will wipe all existing BCORrels if OCC=0.0 atom(s) is/are involved. This can be' switched off via the '-bcorrel_nowipe_zeroocc' flag.
 We will wipe all existing BCORrels if only non-hydrogen atom(s) with fixed B(iso) are involved. This can be switched off via the '-bcorrel_nowipe_fixb_nonhydrogens' flag.
 We will keep all existing BCORrels if hydrogen atom(s) with fixed B(iso) is/are involved. This can be switched off via the '-bcorrel_wipe_fixb_hydrogens' flag.
 No -bcorrel_missing_sigma found. So will set sigma to     2.5 Ang**2 for any bond with no existing BCORrel
 No -bcorrel_missing_sigma_hydrogens found. So will set sigma to     1.0 Ang**2 for any bond with no existing BCORrel involving hydrogens

 No active BCORels found involving OCC=0.0 atom(s)


 No active BCORels found involving fixed-B (only) non-hydrogen atom(s)

    add a default BCOR for bond A|401:C1(CIT) A|401:C2(CIT)
    add a default BCOR for bond A|401:C1(CIT) A|401:O1(CIT)
    add a default BCOR for bond A|401:C1(CIT) A|401:O2(CIT)
    add a default BCOR for bond A|401:C2(CIT) A|401:C3(CIT)
    add a default BCOR for bond A|401:C3(CIT) A|401:C4(CIT)
    add a default BCOR for bond A|401:C3(CIT) A|401:C6(CIT)
    add a default BCOR for bond A|401:C3(CIT) A|401:O7(CIT)
    add a default BCOR for bond A|401:C4(CIT) A|401:C5(CIT)
    add a default BCOR for bond A|401:C5(CIT) A|401:O3(CIT)
    add a default BCOR for bond A|401:C5(CIT) A|401:O4(CIT)
    add a default BCOR for bond A|401:C6(CIT) A|401:O5(CIT)
    add a default BCOR for bond A|401:C6(CIT) A|401:O6(CIT)
    add a default BCOR for bond A|501:C01(LIG) A|501:N02(LIG)
    add a default BCOR for bond A|501:C01(LIG) A|501:C09(LIG)
    add a default BCOR for bond A|501:N02(LIG) A|501:C03(LIG)
    add a default BCOR for bond A|501:C03(LIG) A|501:C04(LIG)
    add a default BCOR for bond A|501:C03(LIG) A|501:N08(LIG)
    add a default BCOR for bond A|501:C04(LIG) A|501:C05(LIG)
    add a default BCOR for bond A|501:C05(LIG) A|501:C06(LIG)
    add a default BCOR for bond A|501:C06(LIG) A|501:C07(LIG)
    add a default BCOR for bond A|501:C07(LIG) A|501:N08(LIG)
    add a default BCOR for bond A|501:C09(LIG) A|501:C10(LIG)
    add a default BCOR for bond A|501:C09(LIG) A|501:C13(LIG)
    add a default BCOR for bond A|501:C10(LIG) A|501:C11(LIG)
    add a default BCOR for bond A|501:C11(LIG) A|501:N12(LIG)
    add a default BCOR for bond A|501:N12(LIG) A|501:C13(LIG)
    add a default BCOR for bond B|401:C1(CIT) B|401:C2(CIT)
    add a default BCOR for bond B|401:C1(CIT) B|401:O1(CIT)
    add a default BCOR for bond B|401:C1(CIT) B|401:O2(CIT)
    add a default BCOR for bond B|401:C2(CIT) B|401:C3(CIT)
    add a default BCOR for bond B|401:C3(CIT) B|401:C4(CIT)
    add a default BCOR for bond B|401:C3(CIT) B|401:C6(CIT)
    add a default BCOR for bond B|401:C3(CIT) B|401:O7(CIT)
    add a default BCOR for bond B|401:C4(CIT) B|401:C5(CIT)
    add a default BCOR for bond B|401:C5(CIT) B|401:O3(CIT)
    add a default BCOR for bond B|401:C5(CIT) B|401:O4(CIT)
    add a default BCOR for bond B|401:C6(CIT) B|401:O5(CIT)
    add a default BCOR for bond B|401:C6(CIT) B|401:O6(CIT)

 Have added a total of        38 BCORrels for bonds which do not already have a BCORrel


 No bonds (for potential BCORrel addition) were skipped due to both atoms having constant/fixed B(iso)


 ----------- Deactivate hydrogen-free planes if hydrogen atoms present

 A total of       0 planes have been deactivated because they are duplicated hydrogen-free and with-hydrogen forms.

 ----------- Handle peptide omega angle(s)

      Torsion angles split into peptide omega and other
         Number of peptide omega angles is       698
         Number of other torsion is             1275
      Peptide omega angles are monitored rather than directly restrained (PLANE used instead).

 ----------- Fix cis-PRO

 Now apply the fix to get correct restraints for cis PRO residues
   Have got      11 bonds records and      26 angles
   from GEOMETRY cards for the special PROC residue
   Weight for ideal-distance contact term=     4.000

 ----------- Prepare SSBOND/LINK for output PDB file


 ----------- Prepare for QM


 ----------- Prepare symmetry in utility distance restraints


 ############### Setting up Rhofit contacts (if needed)

 No ligand-(fixed protein) contact term will be used

 ############### Working out initial function and gradient


   MaxLik Scaling Cycle 0002
 
 Initial functional value = 3.943380E+05 rms gradient=      42.7076
 X-ray contribution to function value = 3.511526E+05
 Geometry contribution (inc ncs)      =      43185.3
 Geometry function summary
     term                           number     weight       rms        GooF      function     fn/numb
     ================================================================================================
     BOND bond lengths (angs)       5668       2.000      0.0084       0.419      1994.38       0.352
     ANGL bond angles (degs)        7691       2.000        1.05       0.625      6017.32       0.782
     TORS split-harmonic tors       1275       0.000       19.09       1.200         0.00       0.000
     SINTOR sinusoidal tors         1275       2.000       19.09                  2711.32       2.127
     PLAN general plane (angs)       967       5.000      0.0141       0.704      2399.35       2.481
     BCORrelations (angs^2)         5668      10.000       1.146       0.409      9485.15       1.673
     CONT (bad contacts)               0       5.000                                 0.00
     IDEAL(ideal-dist contact)      5663       4.000                             13154.15
     CHIRAL (gelly semiharmon)       740       5.000                                50.55
     SIM similarity restraint for NCS and/or TARGET                               7373.14
     OCCSUM restraint on sum of occupanies                                           0.00       as none defined
     DISTAN utility restraints interatomic distance                                  0.00       as none defined
     UTILANGLE utility angle restraints                                              0.00       as none defined
     UTILTOR utility restraints on torsion angles                                    0.00       as none defined
     Peptide omega torsion angles                           2.88 degs - monitor only

 Weighted rms      0.0084         1.050  (temporary rms bond and angle for BUSTER to pick up!)
 Weighted intra-residual rms(bond) for StdProtein (excluding H)      0.0082
 Weighted intra-residual rms(bond) for Buffer     (excluding H)      0.0032

 ############### Running initial check


 Initial Geometry Sanity Check

 Check for duplicate bonds: no duplicates found
 
 Check for duplicate or missing bond angles: all are OK
 Check for bond between an atom and itself not involving symmetry:  all are OK
   There are no bad contacts where Delta/sigma is greater than     6.5
   Worst contact dist=   3.028 std=   3.802 sigma=   0.206 abs(Delta/sigma)=    3.759 ats A|104:CE (MET) B|23:CB (PRO)
   It is likely that the geometry libraries, sequence info and links match the input molecule
 GEOMETRY CHECK OVERALL:  PASS


 ############### Creating geometry/screen report


 ############### Option "tao" - run Tao optimizer

 TAO: Toolkit for Advanced Optimization. Reference: 
     Munson, T., Sarich, J., Wild, S., Benson, S., McInnes, L.C. (2018)
     "TAO User Manual (Revision 3.9)", Mathematics and Computer Science
     Division, Argonne National Laboratory
     ANL/MCS-TM-322, http://www.mcs.anl.gov/petsc/
 
 Have found -keeppdb flag so will write out a .pdb file every 1000000 its during opt
 Will apply TAO optimization 
 Maximum number of iterations      100
 Convergence test on Grms       4.0000 (-glim)
 Limit on rms displacement to original position (rmsD2init): NONE test inactive (-dlim)

    analysing argument no 0 : -autoncs with j=0
    analysing argument no 1 : -hydrogens_input_occ2zero with j=0
    analysing argument no 2 : -hydrogens_output_occ2heavy with j=0
    analysing argument no 3 : -screen with j=0
    analysing argument no 4 : 100 with j=0
    analysing argument no 5 : -screen_sigma with j=0
    analysing argument no 6 : 3.0 with j=0
    analysing argument no 7 : -keeppdb with j=0
    analysing argument no 8 : 1000000 with j=0
    analysing argument no 9 : -taomonitor_verbose with j=0
    found argument starting with "-tao" : -taomonitor_verbose
    analysing argument no 10 : 2 with j=1
    found value after -tao/-options_/-viewer_ argument : "2"
 PETSC/TAO: setting option "-taomonitor_verbose" = "2"


   MaxLik Scaling Cycle 0003
   Iters     Tot_Funct    Xray_Funct    Geom_Funct          Grms     rmsD2init     maxD2init             R         Rfree           LLG       LLGfree     rms(bond)    rms(angle)       Opttag_H
   Iters             K   B_IMPF_FRAG        K_MISS   B_TEMP_MISS   K_IMPF_MISS   B_IMPF_MISS        K_SOLV   B_TEMP_SOLV   K_IMPF_SOLV   B_IMPF_SOLV        B_TEMP     B_TEMP_11     B_TEMP_12     B_TEMP_22     B_TEMP_13     B_TEMP_23     B_TEMP_33      K_WILSON      B_WILSON      FGCYCLES       Scatag_H
   Iters          BOND         ANGLE      IMPROPER       TORSION    SINTORSION      TRIGONAL        PLANAR  BCORRELATION       CONTACT     IDEALDIST        CHIRAL    SIMILARITY       OCCSSUM  UTILDISTANCE     UTILANGLE   UTILTORSION        RHOFIT        ADP-01        ADP-02        ADP-03        ADP-04        ADP-05        ADP-06        ADP-07        ADP-08        ADP-09        ADP-10      RHOFITPC            QM       Funtag_H
       0  3.942763E+05  3.511602E+05       43116.1       42.9514  0.000000E+00  0.000000E+00      0.183459      0.241249  0.000000E+00  0.000000E+00    0.00840993       1.04971       Opttag_D
       0       82.6300      0.949712       1.00000  0.000000E+00       1.00000  0.000000E+00      0.945945       99.6163       1.00000       34.7719  0.000000E+00      -1.29522  0.000000E+00      -1.29522  0.000000E+00  0.000000E+00       2.59044       83.1364     -0.105908        2       Scatag_D
       0       1994.38       6017.32  0.000000E+00  0.000000E+00       2711.32  0.000000E+00       2399.35       9415.93  0.000000E+00       13154.1       50.5490       7373.14 -1.000000E+10 -1.000000E+10 -1.000000E+10 -1.000000E+10 -1.000000E+10  0.000000E+00  0.000000E+00  0.000000E+00  0.000000E+00  0.000000E+00  0.000000E+00  0.000000E+00  0.000000E+00  0.000000E+00  0.000000E+00  0.000000E+00  0.000000E+00       Funtag_D
   Geometry Restraint Deviations SCREEN Information

 

 An atom has moved more than     1.50 A from the initial/last
 position - if this was an accepted trial point, probably need to update the
 non-bonded contact list.


   MaxLik Scaling Cycle 0004

   MaxLik Scaling Cycle 0005

   MaxLik Scaling Cycle 0006

   Wilson plot

   Statistics in resolution bins after refinement cycle 0001
```


|  |  |
| --- | --- |
|  | - Rxpct factors plot- Rxpct factors table- <Log-Lik Gain>- Ln Sigma A- Correlation Coefficients |


```
   Iters     Tot_Funct    Xray_Funct    Geom_Funct          Grms     rmsD2init     maxD2init             R         Rfree           LLG       LLGfree     rms(bond)    rms(angle)
   Iters             K   B_IMPF_FRAG        K_MISS   B_TEMP_MISS   K_IMPF_MISS   B_IMPF_MISS        K_SOLV   B_TEMP_SOLV   K_IMPF_SOLV   B_IMPF_SOLV        B_TEMP     B_TEMP_11     B_TEMP_12     B_TEMP_22     B_TEMP_13     B_TEMP_23     B_TEMP_33      K_WILSON      B_WILSON      FGCYCLES
   Iters          BOND         ANGLE      IMPROPER       TORSION    SINTORSION      TRIGONAL        PLANAR  BCORRELATION       CONTACT     IDEALDIST        CHIRAL    SIMILARITY       OCCSSUM  UTILDISTANCE     UTILANGLE   UTILTORSION        RHOFIT        ADP-01        ADP-02        ADP-03        ADP-04        ADP-05        ADP-06        ADP-07        ADP-08        ADP-09        ADP-10      RHOFITPC            QM
       1  3.939284E+05  3.511761E+05       42752.4       33.1408  3.537344E-04    0.00646289      0.183431      0.241491  2.615076E-04   -0.00136342    0.00838326       1.04959       Opttag_D
       1       82.6302      0.950303       1.00000  0.000000E+00       1.00000  0.000000E+00      0.945960       99.6025       1.00000       34.7572  0.000000E+00      -1.29485  0.000000E+00      -1.29485  0.000000E+00  0.000000E+00       2.58970       83.1364     -0.105908        3       Scatag_D
       1       1981.75       6015.94  0.000000E+00  0.000000E+00       2711.35  0.000000E+00       2397.75       9095.28  0.000000E+00       13150.3       50.9643       7349.08 -1.000000E+10 -1.000000E+10 -1.000000E+10 -1.000000E+10 -1.000000E+10  0.000000E+00  0.000000E+00  0.000000E+00  0.000000E+00  0.000000E+00  0.000000E+00  0.000000E+00  0.000000E+00  0.000000E+00  0.000000E+00  0.000000E+00  0.000000E+00       Funtag_D
   Geometry Restraint Deviations SCREEN Information

 

   MaxLik Scaling Cycle 0007

   MaxLik Scaling Cycle 0008

   Wilson plot

   Statistics in resolution bins after refinement cycle 0002
```


|  |  |
| --- | --- |
|  | - Rxpct factors plot- Rxpct factors table- <Log-Lik Gain>- Ln Sigma A- Correlation Coefficients |


```
   Iters     Tot_Funct    Xray_Funct    Geom_Funct          Grms     rmsD2init     maxD2init             R         Rfree           LLG       LLGfree     rms(bond)    rms(angle)
   Iters             K   B_IMPF_FRAG        K_MISS   B_TEMP_MISS   K_IMPF_MISS   B_IMPF_MISS        K_SOLV   B_TEMP_SOLV   K_IMPF_SOLV   B_IMPF_SOLV        B_TEMP     B_TEMP_11     B_TEMP_12     B_TEMP_22     B_TEMP_13     B_TEMP_23     B_TEMP_33      K_WILSON      B_WILSON      FGCYCLES
   Iters          BOND         ANGLE      IMPROPER       TORSION    SINTORSION      TRIGONAL        PLANAR  BCORRELATION       CONTACT     IDEALDIST        CHIRAL    SIMILARITY       OCCSSUM  UTILDISTANCE     UTILANGLE   UTILTORSION        RHOFIT        ADP-01        ADP-02        ADP-03        ADP-04        ADP-05        ADP-06        ADP-07        ADP-08        ADP-09        ADP-10      RHOFITPC            QM
       2  3.937807E+05  3.511920E+05       42588.7       37.8750  9.365149E-04     0.0193791      0.183432      0.241483  2.167880E-04   -0.00130100    0.00836329       1.05130       Opttag_D
       2       82.6301      0.951081       1.00000  0.000000E+00       1.00000  0.000000E+00      0.945972       99.5865       1.00000       34.7279  0.000000E+00      -1.29503  0.000000E+00      -1.29503  0.000000E+00  0.000000E+00       2.59005       83.1364     -0.105908        2       Scatag_D
       2       1972.32       6035.56  0.000000E+00  0.000000E+00       2711.13  0.000000E+00       2395.38       8969.78  0.000000E+00       13143.1       51.4665       7309.99 -1.000000E+10 -1.000000E+10 -1.000000E+10 -1.000000E+10 -1.000000E+10  0.000000E+00  0.000000E+00  0.000000E+00  0.000000E+00  0.000000E+00  0.000000E+00  0.000000E+00  0.000000E+00  0.000000E+00  0.000000E+00  0.000000E+00  0.000000E+00       Funtag_D
   Geometry Restraint Deviations SCREEN Information

 
       3  3.936538E+05  3.512039E+05       42449.9       28.4786    0.00140491     0.0304124      0.183429      0.241474  1.831468E-04   -0.00124533    0.00834304       1.04960       Opttag_D
       3       82.6301      0.951697       1.00000  0.000000E+00       1.00000  0.000000E+00      0.945984       99.5726       1.00000       34.7036  0.000000E+00      -1.29511  0.000000E+00      -1.29511  0.000000E+00  0.000000E+00       2.59022       83.1364     -0.105908        1       Scatag_D
       3       1962.78       6015.98  0.000000E+00  0.000000E+00       2711.17  0.000000E+00       2393.66       8893.11  0.000000E+00       13138.7       51.7214       7282.71 -1.000000E+10 -1.000000E+10 -1.000000E+10 -1.000000E+10 -1.000000E+10  0.000000E+00  0.000000E+00  0.000000E+00  0.000000E+00  0.000000E+00  0.000000E+00  0.000000E+00  0.000000E+00  0.000000E+00  0.000000E+00  0.000000E+00  0.000000E+00       Funtag_D
       4  3.935574E+05  3.512196E+05       42337.8       26.1147    0.00204416     0.0451829      0.183431      0.241468  1.391387E-04   -0.00119414    0.00832841       1.04917       Opttag_D
       4       82.6298      0.952455       1.00000  0.000000E+00       1.00000  0.000000E+00      0.946001       99.5530       1.00000       34.6723  0.000000E+00      -1.29531  0.000000E+00      -1.29531  0.000000E+00  0.000000E+00       2.59062       83.1364     -0.105908        1       Scatag_D
       4       1955.90       6011.14  0.000000E+00  0.000000E+00       2711.10  0.000000E+00       2391.86       8829.84  0.000000E+00       13132.9       51.8828       7253.22 -1.000000E+10 -1.000000E+10 -1.000000E+10 -1.000000E+10 -1.000000E+10  0.000000E+00  0.000000E+00  0.000000E+00  0.000000E+00  0.000000E+00  0.000000E+00  0.000000E+00  0.000000E+00  0.000000E+00  0.000000E+00  0.000000E+00  0.000000E+00       Funtag_D
       5  3.934737E+05  3.512350E+05       42238.7       26.9386    0.00280595     0.0625723      0.183429      0.241463  9.584206E-05   -0.00114995    0.00831891       1.04945       Opttag_D
       5       82.6294      0.953217       1.00000  0.000000E+00       1.00000  0.000000E+00      0.946024       99.5270       1.00000       34.6360  0.000000E+00      -1.29576  0.000000E+00      -1.29576  0.000000E+00  0.000000E+00       2.59152       83.1364     -0.105908        1       Scatag_D
       5       1951.44       6014.24  0.000000E+00  0.000000E+00       2710.83  0.000000E+00       2390.24       8766.88  0.000000E+00       13127.0       51.8381       7226.22 -1.000000E+10 -1.000000E+10 -1.000000E+10 -1.000000E+10 -1.000000E+10  0.000000E+00  0.000000E+00  0.000000E+00  0.000000E+00  0.000000E+00  0.000000E+00  0.000000E+00  0.000000E+00  0.000000E+00  0.000000E+00  0.000000E+00  0.000000E+00       Funtag_D
       6  3.933903E+05  3.512443E+05       42146.0       23.7365    0.00357377     0.0793353      0.183426      0.241457  6.951852E-05   -0.00114234    0.00830732       1.04900       Opttag_D
       6       82.6288      0.953758       1.00000  0.000000E+00       1.00000  0.000000E+00      0.946050       99.4987       1.00000       34.6001  0.000000E+00      -1.29648  0.000000E+00      -1.29648  0.000000E+00  0.000000E+00       2.59297       83.1364     -0.105908        1       Scatag_D
       6       1946.01       6009.15  0.000000E+00  0.000000E+00       2710.52  0.000000E+00       2388.90       8713.28  0.000000E+00       13121.7       51.5801       7204.81 -1.000000E+10 -1.000000E+10 -1.000000E+10 -1.000000E+10 -1.000000E+10  0.000000E+00  0.000000E+00  0.000000E+00  0.000000E+00  0.000000E+00  0.000000E+00  0.000000E+00  0.000000E+00  0.000000E+00  0.000000E+00  0.000000E+00  0.000000E+00       Funtag_D
       7  3.933188E+05  3.512456E+05       42073.2       24.1806    0.00436505     0.0942243      0.183421      0.241450  6.585965E-05   -0.00115876    0.00829750       1.04903       Opttag_D
       7       82.6278      0.954026       1.00000  0.000000E+00       1.00000  0.000000E+00      0.946079       99.4686       1.00000       34.5629  0.000000E+00      -1.29745  0.000000E+00      -1.29745  0.000000E+00  0.000000E+00       2.59491       83.1364     -0.105908        1       Scatag_D
       7       1941.41       6009.45  0.000000E+00  0.000000E+00       2710.06  0.000000E+00       2387.46       8670.00  0.000000E+00       13116.2       51.2040       7187.38 -1.000000E+10 -1.000000E+10 -1.000000E+10 -1.000000E+10 -1.000000E+10  0.000000E+00  0.000000E+00  0.000000E+00  0.000000E+00  0.000000E+00  0.000000E+00  0.000000E+00  0.000000E+00  0.000000E+00  0.000000E+00  0.000000E+00  0.000000E+00       Funtag_D
       8  3.932607E+05  3.512394E+05       42021.3       21.7669    0.00485455      0.100943      0.183416      0.241445  8.323928E-05   -0.00118702    0.00829295       1.04839       Opttag_D
       8       82.6271      0.953972       1.00000  0.000000E+00       1.00000  0.000000E+00      0.946100       99.4493       1.00000       34.5391  0.000000E+00      -1.29811  0.000000E+00      -1.29811  0.000000E+00  0.000000E+00       2.59622       83.1364     -0.105908        1       Scatag_D
       8       1939.28       6002.19  0.000000E+00  0.000000E+00       2709.68  0.000000E+00       2386.31       8641.47  0.000000E+00       13113.6       50.9701       7177.81 -1.000000E+10 -1.000000E+10 -1.000000E+10 -1.000000E+10 -1.000000E+10  0.000000E+00  0.000000E+00  0.000000E+00  0.000000E+00  0.000000E+00  0.000000E+00  0.000000E+00  0.000000E+00  0.000000E+00  0.000000E+00  0.000000E+00  0.000000E+00       Funtag_D
       9  3.932001E+05  3.512288E+05       41971.4       20.1434    0.00540850      0.106245      0.183407      0.241435  1.133233E-04   -0.00122734    0.00828755       1.04798       Opttag_D
       9       82.6262      0.953803       1.00000  0.000000E+00       1.00000  0.000000E+00      0.946125       99.4282       1.00000       34.5114  0.000000E+00      -1.29879  0.000000E+00      -1.29879  0.000000E+00  0.000000E+00       2.59758       83.1364     -0.105908        1       Scatag_D
       9       1936.76       5997.52  0.000000E+00  0.000000E+00       2709.32  0.000000E+00       2384.83       8612.22  0.000000E+00       13110.9       50.8018       7168.99 -1.000000E+10 -1.000000E+10 -1.000000E+10 -1.000000E+10 -1.000000E+10  0.000000E+00  0.000000E+00  0.000000E+00  0.000000E+00  0.000000E+00  0.000000E+00  0.000000E+00  0.000000E+00  0.000000E+00  0.000000E+00  0.000000E+00  0.000000E+00       Funtag_D
      10  3.931281E+05  3.512134E+05       41914.7       23.1167    0.00651124      0.114597      0.183392      0.241415  1.566199E-04   -0.00128494    0.00828470       1.04809       Opttag_D
      10       82.6250      0.953614       1.00000  0.000000E+00       1.00000  0.000000E+00      0.946173       99.3863       1.00000       34.4587  0.000000E+00      -1.29999  0.000000E+00      -1.29999  0.000000E+00  0.000000E+00       2.59998       83.1364     -0.105908        1       Scatag_D
      10       1935.43       5998.70  0.000000E+00  0.000000E+00       2708.62  0.000000E+00       2382.18       8577.10  0.000000E+00       13106.0       50.7201       7156.04 -1.000000E+10 -1.000000E+10 -1.000000E+10 -1.000000E+10 -1.000000E+10  0.000000E+00  0.000000E+00  0.000000E+00  0.000000E+00  0.000000E+00  0.000000E+00  0.000000E+00  0.000000E+00  0.000000E+00  0.000000E+00  0.000000E+00  0.000000E+00       Funtag_D
      11  3.930684E+05  3.512057E+05       41862.7       24.7496    0.00778321      0.124444      0.183382      0.241393  1.781666E-04   -0.00130583    0.00828273       1.04825       Opttag_D
      11       82.6243      0.953659       1.00000  0.000000E+00       1.00000  0.000000E+00      0.946228       99.3347       1.00000       34.4022  0.000000E+00      -1.30111  0.000000E+00      -1.30111  0.000000E+00  0.000000E+00       2.60222       83.1364     -0.105908        1       Scatag_D
      11       1934.51       6000.54  0.000000E+00  0.000000E+00       2707.95  0.000000E+00       2379.76       8544.80  0.000000E+00       13101.5       50.6683       7142.91 -1.000000E+10 -1.000000E+10 -1.000000E+10 -1.000000E+10 -1.000000E+10  0.000000E+00  0.000000E+00  0.000000E+00  0.000000E+00  0.000000E+00  0.000000E+00  0.000000E+00  0.000000E+00  0.000000E+00  0.000000E+00  0.000000E+00  0.000000E+00       Funtag_D
      12  3.930133E+05  3.512021E+05       41811.2       18.4182    0.00834930      0.126926      0.183375      0.241382  1.883302E-04   -0.00131730    0.00827887       1.04729       Opttag_D
      12       82.6243      0.953687       1.00000  0.000000E+00       1.00000  0.000000E+00      0.946254       99.3112       1.00000       34.3759  0.000000E+00      -1.30167  0.000000E+00      -1.30167  0.000000E+00  0.000000E+00       2.60334       83.1364     -0.105908        1       Scatag_D
      12       1932.70       5989.52  0.000000E+00  0.000000E+00       2707.66  0.000000E+00       2378.58       8518.62  0.000000E+00       13098.9       50.7052       7134.45 -1.000000E+10 -1.000000E+10 -1.000000E+10 -1.000000E+10 -1.000000E+10  0.000000E+00  0.000000E+00  0.000000E+00  0.000000E+00  0.000000E+00  0.000000E+00  0.000000E+00  0.000000E+00  0.000000E+00  0.000000E+00  0.000000E+00  0.000000E+00       Funtag_D
      13  3.929594E+05  3.511982E+05       41761.3       17.1234    0.00923790      0.130206      0.183368      0.241366  1.993068E-04   -0.00132865    0.00827781       1.04694       Opttag_D
      13       82.6244      0.953760       1.00000  0.000000E+00       1.00000  0.000000E+00      0.946293       99.2738       1.00000       34.3362  0.000000E+00      -1.30253  0.000000E+00      -1.30253  0.000000E+00  0.000000E+00       2.60505       83.1364     -0.105908        1       Scatag_D
      13       1932.21       5985.56  0.000000E+00  0.000000E+00       2707.15  0.000000E+00       2376.99       8492.97  0.000000E+00       13094.6       50.7363       7121.07 -1.000000E+10 -1.000000E+10 -1.000000E+10 -1.000000E+10 -1.000000E+10  0.000000E+00  0.000000E+00  0.000000E+00  0.000000E+00  0.000000E+00  0.000000E+00  0.000000E+00  0.000000E+00  0.000000E+00  0.000000E+00  0.000000E+00  0.000000E+00       Funtag_D
      14  3.929252E+05  3.511928E+05       41732.4       23.1484     0.0103788      0.133511      0.183359      0.241344  2.144504E-04   -0.00133408    0.00828410       1.04740       Opttag_D
      14       82.6249      0.953800       1.00000  0.000000E+00       1.00000  0.000000E+00      0.946344       99.2260       1.00000       34.2885  0.000000E+00      -1.30351  0.000000E+00      -1.30351  0.000000E+00  0.000000E+00       2.60701       83.1364     -0.105908        1       Scatag_D
      14       1935.15       5990.80  0.000000E+00  0.000000E+00       2706.44  0.000000E+00       2375.33       8476.94  0.000000E+00       13090.3       50.7353       7106.72 -1.000000E+10 -1.000000E+10 -1.000000E+10 -1.000000E+10 -1.000000E+10  0.000000E+00  0.000000E+00  0.000000E+00  0.000000E+00  0.000000E+00  0.000000E+00  0.000000E+00  0.000000E+00  0.000000E+00  0.000000E+00  0.000000E+00  0.000000E+00       Funtag_D
      15  3.928801E+05  3.511880E+05       41692.1       17.3387     0.0110928      0.144808      0.183352      0.241329  2.277646E-04   -0.00133855    0.00828146       1.04646       Opttag_D
      15       82.6254      0.953772       1.00000  0.000000E+00       1.00000  0.000000E+00      0.946375       99.1974       1.00000       34.2596  0.000000E+00      -1.30401  0.000000E+00      -1.30401  0.000000E+00  0.000000E+00       2.60803       83.1364     -0.105908        1       Scatag_D
      15       1933.91       5980.04  0.000000E+00  0.000000E+00       2706.00  0.000000E+00       2374.39       8461.88  0.000000E+00       13087.0       50.7128       7098.12 -1.000000E+10 -1.000000E+10 -1.000000E+10 -1.000000E+10 -1.000000E+10  0.000000E+00  0.000000E+00  0.000000E+00  0.000000E+00  0.000000E+00  0.000000E+00  0.000000E+00  0.000000E+00  0.000000E+00  0.000000E+00  0.000000E+00  0.000000E+00       Funtag_D
      16  3.928222E+05  3.511765E+05       41645.7       15.8138     0.0122743      0.164005      0.183342      0.241299  2.602879E-04   -0.00132696    0.00828176       1.04634       Opttag_D
      16       82.6264      0.953618       1.00000  0.000000E+00       1.00000  0.000000E+00      0.946425       99.1524       1.00000       34.2131  0.000000E+00      -1.30469  0.000000E+00      -1.30469  0.000000E+00  0.000000E+00       2.60937       83.1364     -0.105908        1       Scatag_D
      16       1934.05       5978.71  0.000000E+00  0.000000E+00       2705.20  0.000000E+00       2373.02       8437.21  0.000000E+00       13081.8       50.6722       7085.01 -1.000000E+10 -1.000000E+10 -1.000000E+10 -1.000000E+10 -1.000000E+10  0.000000E+00  0.000000E+00  0.000000E+00  0.000000E+00  0.000000E+00  0.000000E+00  0.000000E+00  0.000000E+00  0.000000E+00  0.000000E+00  0.000000E+00  0.000000E+00       Funtag_D
      17  3.927851E+05  3.511641E+05       41621.1       22.0452     0.0137351      0.187979      0.183330      0.241256  2.952505E-04   -0.00127914    0.00828869       1.04702       Opttag_D
      17       82.6283      0.953505       1.00000  0.000000E+00       1.00000  0.000000E+00      0.946491       99.0985       1.00000       34.1576  0.000000E+00      -1.30522  0.000000E+00      -1.30522  0.000000E+00  0.000000E+00       2.61045       83.1364     -0.105908        1       Scatag_D
      17       1937.29       5986.46  0.000000E+00  0.000000E+00       2704.28  0.000000E+00       2371.66       8421.04  0.000000E+00       13076.9       50.6413       7072.78 -1.000000E+10 -1.000000E+10 -1.000000E+10 -1.000000E+10 -1.000000E+10  0.000000E+00  0.000000E+00  0.000000E+00  0.000000E+00  0.000000E+00  0.000000E+00  0.000000E+00  0.000000E+00  0.000000E+00  0.000000E+00  0.000000E+00  0.000000E+00       Funtag_D
      18  3.927451E+05  3.511534E+05       41591.7       15.3911     0.0145464      0.201904      0.183322      0.241228  3.252329E-04   -0.00124111    0.00828716       1.04594       Opttag_D
      18       82.6294      0.953344       1.00000  0.000000E+00       1.00000  0.000000E+00      0.946526       99.0710       1.00000       34.1269  0.000000E+00      -1.30545  0.000000E+00      -1.30545  0.000000E+00  0.000000E+00       2.61089       83.1364     -0.105908        1       Scatag_D
      18       1936.58       5974.10  0.000000E+00  0.000000E+00       2703.71  0.000000E+00       2371.11       8415.44  0.000000E+00       13073.9       50.6428       7066.17 -1.000000E+10 -1.000000E+10 -1.000000E+10 -1.000000E+10 -1.000000E+10  0.000000E+00  0.000000E+00  0.000000E+00  0.000000E+00  0.000000E+00  0.000000E+00  0.000000E+00  0.000000E+00  0.000000E+00  0.000000E+00  0.000000E+00  0.000000E+00       Funtag_D
      19  3.927087E+05  3.511386E+05       41570.1       14.0093     0.0151972      0.214228      0.183311      0.241198  3.668017E-04   -0.00118255    0.00828658       1.04565       Opttag_D
      19       82.6303      0.953105       1.00000  0.000000E+00       1.00000  0.000000E+00      0.946557       99.0525       1.00000       34.1001  0.000000E+00      -1.30553  0.000000E+00      -1.30553  0.000000E+00  0.000000E+00       2.61105       83.1364     -0.105908        1       Scatag_D
      19       1936.31       5970.82  0.000000E+00  0.000000E+00       2703.26  0.000000E+00       2370.69       8406.63  0.000000E+00       13071.4       50.6389       7060.33 -1.000000E+10 -1.000000E+10 -1.000000E+10 -1.000000E+10 -1.000000E+10  0.000000E+00  0.000000E+00  0.000000E+00  0.000000E+00  0.000000E+00  0.000000E+00  0.000000E+00  0.000000E+00  0.000000E+00  0.000000E+00  0.000000E+00  0.000000E+00       Funtag_D
      20  3.926711E+05  3.511178E+05       41553.3       16.8268     0.0161709      0.232932      0.183295      0.241157  4.253436E-04   -0.00109018    0.00828864       1.04576       Opttag_D
      20       82.6317      0.952771       1.00000  0.000000E+00       1.00000  0.000000E+00      0.946602       99.0248       1.00000       34.0606  0.000000E+00      -1.30569  0.000000E+00      -1.30569  0.000000E+00  0.000000E+00       2.61138       83.1364     -0.105908        1       Scatag_D
      20       1937.26       5972.08  0.000000E+00  0.000000E+00       2702.57  0.000000E+00       2370.36       8399.43  0.000000E+00       13068.2       50.6505       7052.70 -1.000000E+10 -1.000000E+10 -1.000000E+10 -1.000000E+10 -1.000000E+10  0.000000E+00  0.000000E+00  0.000000E+00  0.000000E+00  0.000000E+00  0.000000E+00  0.000000E+00  0.000000E+00  0.000000E+00  0.000000E+00  0.000000E+00  0.000000E+00       Funtag_D
      21  3.926309E+05  3.510947E+05       41536.2       16.4850     0.0173924      0.256374      0.183276      0.241114  4.903902E-04 -9.822374E-04    0.00829038       1.04546       Opttag_D
      21       82.6331      0.952398       1.00000  0.000000E+00       1.00000  0.000000E+00      0.946658       98.9899       1.00000       34.0130  0.000000E+00      -1.30598  0.000000E+00      -1.30598  0.000000E+00  0.000000E+00       2.61195       83.1364     -0.105908        1       Scatag_D
      21       1938.08       5968.64  0.000000E+00  0.000000E+00       2701.89  0.000000E+00       2370.24       8397.78  0.000000E+00       13065.0       50.6968       7043.92 -1.000000E+10 -1.000000E+10 -1.000000E+10 -1.000000E+10 -1.000000E+10  0.000000E+00  0.000000E+00  0.000000E+00  0.000000E+00  0.000000E+00  0.000000E+00  0.000000E+00  0.000000E+00  0.000000E+00  0.000000E+00  0.000000E+00  0.000000E+00       Funtag_D
      22  3.925942E+05  3.510756E+05       41518.7       15.3303     0.0184365      0.276879      0.183260      0.241076  5.441552E-04 -8.726035E-04    0.00829121       1.04513       Opttag_D
      22       82.6341      0.952069       1.00000  0.000000E+00       1.00000  0.000000E+00      0.946706       98.9593       1.00000       33.9733  0.000000E+00      -1.30625  0.000000E+00      -1.30625  0.000000E+00  0.000000E+00       2.61251       83.1364     -0.105908        1       Scatag_D
      22       1938.47       5964.91  0.000000E+00  0.000000E+00       2701.20  0.000000E+00       2370.29       8395.38  0.000000E+00       13062.7       50.7995       7034.94 -1.000000E+10 -1.000000E+10 -1.000000E+10 -1.000000E+10 -1.000000E+10  0.000000E+00  0.000000E+00  0.000000E+00  0.000000E+00  0.000000E+00  0.000000E+00  0.000000E+00  0.000000E+00  0.000000E+00  0.000000E+00  0.000000E+00  0.000000E+00       Funtag_D
      23  3.925601E+05  3.510603E+05       41499.8       13.3693     0.0194213      0.296135      0.183246      0.241050  5.871470E-04 -7.917063E-04    0.00829040       1.04486       Opttag_D
      23       82.6346      0.951790       1.00000  0.000000E+00       1.00000  0.000000E+00      0.946750       98.9296       1.00000       33.9371  0.000000E+00      -1.30656  0.000000E+00      -1.30656  0.000000E+00  0.000000E+00       2.61312       83.1364     -0.105908        1       Scatag_D
      23       1938.09       5961.85  0.000000E+00  0.000000E+00       2700.68  0.000000E+00       2370.36       8392.75  0.000000E+00       13060.4       50.9341       7024.70 -1.000000E+10 -1.000000E+10 -1.000000E+10 -1.000000E+10 -1.000000E+10  0.000000E+00  0.000000E+00  0.000000E+00  0.000000E+00  0.000000E+00  0.000000E+00  0.000000E+00  0.000000E+00  0.000000E+00  0.000000E+00  0.000000E+00  0.000000E+00       Funtag_D
      24  3.925333E+05  3.510483E+05       41485.0       13.2777     0.0202031      0.311647      0.183234      0.241030  6.208899E-04 -7.289203E-04    0.00829184       1.04472       Opttag_D
      24       82.6345      0.951557       1.00000  0.000000E+00       1.00000  0.000000E+00      0.946782       98.9066       1.00000       33.9086  0.000000E+00      -1.30679  0.000000E+00      -1.30679  0.000000E+00  0.000000E+00       2.61357       83.1364     -0.105908        1       Scatag_D
      24       1938.76       5960.25  0.000000E+00  0.000000E+00       2700.22  0.000000E+00       2370.56       8390.01  0.000000E+00       13059.0       51.0665       7015.09 -1.000000E+10 -1.000000E+10 -1.000000E+10 -1.000000E+10 -1.000000E+10  0.000000E+00  0.000000E+00  0.000000E+00  0.000000E+00  0.000000E+00  0.000000E+00  0.000000E+00  0.000000E+00  0.000000E+00  0.000000E+00  0.000000E+00  0.000000E+00       Funtag_D
      25  3.925052E+05  3.510338E+05       41471.4       12.9043     0.0212435      0.332179      0.183223      0.241008  6.616456E-04 -6.468157E-04    0.00829231       1.04451       Opttag_D
      25       82.6342      0.951273       1.00000  0.000000E+00       1.00000  0.000000E+00      0.946824       98.8760       1.00000       33.8721  0.000000E+00      -1.30688  0.000000E+00      -1.30688  0.000000E+00  0.000000E+00       2.61377       83.1364     -0.105908        1       Scatag_D
      25       1938.98       5957.79  0.000000E+00  0.000000E+00       2699.63  0.000000E+00       2371.29       8392.58  0.000000E+00       13057.3       51.2111       7002.54 -1.000000E+10 -1.000000E+10 -1.000000E+10 -1.000000E+10 -1.000000E+10  0.000000E+00  0.000000E+00  0.000000E+00  0.000000E+00  0.000000E+00  0.000000E+00  0.000000E+00  0.000000E+00  0.000000E+00  0.000000E+00  0.000000E+00  0.000000E+00       Funtag_D
      26  3.924779E+05  3.510232E+05       41454.7       12.0827     0.0221453      0.349625      0.183211      0.240993  6.914248E-04 -5.839090E-04    0.00829357       1.04428       Opttag_D
      26       82.6339      0.951089       1.00000  0.000000E+00       1.00000  0.000000E+00      0.946857       98.8514       1.00000       33.8416  0.000000E+00      -1.30675  0.000000E+00      -1.30675  0.000000E+00  0.000000E+00       2.61350       83.1364     -0.105908        1       Scatag_D
      26       1939.57       5955.19  0.000000E+00  0.000000E+00       2699.16  0.000000E+00       2372.35       8388.56  0.000000E+00       13055.9       51.2530       6992.72 -1.000000E+10 -1.000000E+10 -1.000000E+10 -1.000000E+10 -1.000000E+10  0.000000E+00  0.000000E+00  0.000000E+00  0.000000E+00  0.000000E+00  0.000000E+00  0.000000E+00  0.000000E+00  0.000000E+00  0.000000E+00  0.000000E+00  0.000000E+00       Funtag_D
      27  3.924497E+05  3.510098E+05       41439.8       12.5494     0.0234541      0.374449      0.183198      0.240971  7.291315E-04 -4.997518E-04    0.00829389       1.04410       Opttag_D
      27       82.6334      0.950876       1.00000  0.000000E+00       1.00000  0.000000E+00      0.946903       98.8172       1.00000       33.7995  0.000000E+00      -1.30636  0.000000E+00      -1.30636  0.000000E+00  0.000000E+00       2.61272       83.1364     -0.105908        1       Scatag_D
      27       1939.72       5953.13  0.000000E+00  0.000000E+00       2698.35  0.000000E+00       2374.15       8389.07  0.000000E+00       13054.5       51.2590       6979.66 -1.000000E+10 -1.000000E+10 -1.000000E+10 -1.000000E+10 -1.000000E+10  0.000000E+00  0.000000E+00  0.000000E+00  0.000000E+00  0.000000E+00  0.000000E+00  0.000000E+00  0.000000E+00  0.000000E+00  0.000000E+00  0.000000E+00  0.000000E+00       Funtag_D
      28  3.924278E+05  3.509995E+05       41428.3       12.8862     0.0246924      0.397425      0.183188      0.240951  7.580975E-04 -4.187338E-04    0.00829257       1.04391       Opttag_D
      28       82.6330      0.950731       1.00000  0.000000E+00       1.00000  0.000000E+00      0.946946       98.7847       1.00000       33.7618  0.000000E+00      -1.30593  0.000000E+00      -1.30593  0.000000E+00  0.000000E+00       2.61186       83.1364     -0.105908        1       Scatag_D
      28       1939.10       5950.97  0.000000E+00  0.000000E+00       2697.61  0.000000E+00       2375.89       8391.37  0.000000E+00       13053.3       51.2347       6968.76 -1.000000E+10 -1.000000E+10 -1.000000E+10 -1.000000E+10 -1.000000E+10  0.000000E+00  0.000000E+00  0.000000E+00  0.000000E+00  0.000000E+00  0.000000E+00  0.000000E+00  0.000000E+00  0.000000E+00  0.000000E+00  0.000000E+00  0.000000E+00       Funtag_D
      29  3.924074E+05  3.509928E+05       41414.6       11.4509     0.0255067      0.412320      0.183181      0.240944  7.770017E-04 -3.855297E-04    0.00829013       1.04381       Opttag_D
      29       82.6325      0.950632       1.00000  0.000000E+00       1.00000  0.000000E+00      0.946972       98.7639       1.00000       33.7377  0.000000E+00      -1.30563  0.000000E+00      -1.30563  0.000000E+00  0.000000E+00       2.61127       83.1364     -0.105908        1       Scatag_D
      29       1937.96       5949.79  0.000000E+00  0.000000E+00       2697.11  0.000000E+00       2376.95       8387.64  0.000000E+00       13052.3       51.2253       6961.60 -1.000000E+10 -1.000000E+10 -1.000000E+10 -1.000000E+10 -1.000000E+10  0.000000E+00  0.000000E+00  0.000000E+00  0.000000E+00  0.000000E+00  0.000000E+00  0.000000E+00  0.000000E+00  0.000000E+00  0.000000E+00  0.000000E+00  0.000000E+00       Funtag_D
      30  3.923821E+05  3.509823E+05       41399.8       11.1260     0.0268127      0.435923      0.183171      0.240932  8.064759E-04 -3.298676E-04    0.00828810       1.04368       Opttag_D
      30       82.6315      0.950474       1.00000  0.000000E+00       1.00000  0.000000E+00      0.947011       98.7315       1.00000       33.7004  0.000000E+00      -1.30515  0.000000E+00      -1.30515  0.000000E+00  0.000000E+00       2.61031       83.1364     -0.105908        1       Scatag_D
      30       1937.01       5948.36  0.000000E+00  0.000000E+00       2696.18  0.000000E+00       2378.48       8385.71  0.000000E+00       13051.6       51.2704       6951.17 -1.000000E+10 -1.000000E+10 -1.000000E+10 -1.000000E+10 -1.000000E+10  0.000000E+00  0.000000E+00  0.000000E+00  0.000000E+00  0.000000E+00  0.000000E+00  0.000000E+00  0.000000E+00  0.000000E+00  0.000000E+00  0.000000E+00  0.000000E+00       Funtag_D
      31  3.923598E+05  3.509701E+05       41389.6       14.2815     0.0283450      0.463265      0.183159      0.240918  8.408286E-04 -2.789144E-04    0.00828811       1.04401       Opttag_D
      31       82.6300      0.950265       1.00000  0.000000E+00       1.00000  0.000000E+00      0.947053       98.6946       1.00000       33.6590  0.000000E+00      -1.30452  0.000000E+00      -1.30452  0.000000E+00  0.000000E+00       2.60904       83.1364     -0.105908        1       Scatag_D
      31       1937.02       5952.07  0.000000E+00  0.000000E+00       2695.24  0.000000E+00       2379.98       8383.43  0.000000E+00       13051.1       51.3938       6939.39 -1.000000E+10 -1.000000E+10 -1.000000E+10 -1.000000E+10 -1.000000E+10  0.000000E+00  0.000000E+00  0.000000E+00  0.000000E+00  0.000000E+00  0.000000E+00  0.000000E+00  0.000000E+00  0.000000E+00  0.000000E+00  0.000000E+00  0.000000E+00       Funtag_D
      32  3.923329E+05  3.509595E+05       41373.4       12.2681     0.0298393      0.489557      0.183146      0.240904  8.708110E-04 -2.283234E-04    0.00828782       1.04372       Opttag_D
      32       82.6285      0.950081       1.00000  0.000000E+00       1.00000  0.000000E+00      0.947089       98.6607       1.00000       33.6210  0.000000E+00      -1.30385  0.000000E+00      -1.30385  0.000000E+00  0.000000E+00       2.60770       83.1364     -0.105908        1       Scatag_D
      32       1936.88       5948.85  0.000000E+00  0.000000E+00       2694.28  0.000000E+00       2381.37       8380.44  0.000000E+00       13051.2       51.5443       6928.83 -1.000000E+10 -1.000000E+10 -1.000000E+10 -1.000000E+10 -1.000000E+10  0.000000E+00  0.000000E+00  0.000000E+00  0.000000E+00  0.000000E+00  0.000000E+00  0.000000E+00  0.000000E+00  0.000000E+00  0.000000E+00  0.000000E+00  0.000000E+00       Funtag_D
      33  3.923054E+05  3.509486E+05       41356.8       12.1408     0.0313726      0.516226      0.183133      0.240890  9.011999E-04 -1.854599E-04    0.00828923       1.04366       Opttag_D
      33       82.6265      0.949885       1.00000  0.000000E+00       1.00000  0.000000E+00      0.947119       98.6286       1.00000       33.5845  0.000000E+00      -1.30311  0.000000E+00      -1.30311  0.000000E+00  0.000000E+00       2.60623       83.1364     -0.105908        1       Scatag_D
      33       1937.54       5948.07  0.000000E+00  0.000000E+00       2693.24  0.000000E+00       2382.25       8373.86  0.000000E+00       13052.5       51.7038       6917.58 -1.000000E+10 -1.000000E+10 -1.000000E+10 -1.000000E+10 -1.000000E+10  0.000000E+00  0.000000E+00  0.000000E+00  0.000000E+00  0.000000E+00  0.000000E+00  0.000000E+00  0.000000E+00  0.000000E+00  0.000000E+00  0.000000E+00  0.000000E+00       Funtag_D
      34  3.922827E+05  3.509378E+05       41344.9       13.3722     0.0330216      0.544689      0.183121      0.240876  9.318938E-04 -1.458565E-04    0.00829159       1.04377       Opttag_D
      34       82.6241      0.949676       1.00000  0.000000E+00       1.00000  0.000000E+00      0.947149       98.5954       1.00000       33.5481  0.000000E+00      -1.30238  0.000000E+00      -1.30238  0.000000E+00  0.000000E+00       2.60476       83.1364     -0.105908        1       Scatag_D
      34       1938.65       5949.34  0.000000E+00  0.000000E+00       2692.32  0.000000E+00       2382.99       8369.39  0.000000E+00       13054.2       51.8058       6906.23 -1.000000E+10 -1.000000E+10 -1.000000E+10 -1.000000E+10 -1.000000E+10  0.000000E+00  0.000000E+00  0.000000E+00  0.000000E+00  0.000000E+00  0.000000E+00  0.000000E+00  0.000000E+00  0.000000E+00  0.000000E+00  0.000000E+00  0.000000E+00       Funtag_D
      35  3.922611E+05  3.509311E+05       41330.0       11.0919     0.0340128      0.561712      0.183114      0.240867  9.505947E-04 -1.313675E-04    0.00829275       1.04370       Opttag_D
      35       82.6223      0.949561       1.00000  0.000000E+00       1.00000  0.000000E+00      0.947162       98.5783       1.00000       33.5277  0.000000E+00      -1.30198  0.000000E+00      -1.30198  0.000000E+00  0.000000E+00       2.60396       83.1364     -0.105908        1       Scatag_D
      35       1939.19       5948.62  0.000000E+00  0.000000E+00       2691.73  0.000000E+00       2383.05       8361.65  0.000000E+00       13055.3       51.8150       6898.63 -1.000000E+10 -1.000000E+10 -1.000000E+10 -1.000000E+10 -1.000000E+10  0.000000E+00  0.000000E+00  0.000000E+00  0.000000E+00  0.000000E+00  0.000000E+00  0.000000E+00  0.000000E+00  0.000000E+00  0.000000E+00  0.000000E+00  0.000000E+00       Funtag_D
      36  3.922387E+05  3.509232E+05       41315.5       9.82159     0.0348302      0.582452      0.183106      0.240861  9.728528E-04 -1.322126E-04    0.00829292       1.04371       Opttag_D
      36       82.6203      0.949417       1.00000  0.000000E+00       1.00000  0.000000E+00      0.947171       98.5660       1.00000       33.5119  0.000000E+00      -1.30168  0.000000E+00      -1.30168  0.000000E+00  0.000000E+00       2.60336       83.1364     -0.105908        1       Scatag_D
      36       1939.27       5948.66  0.000000E+00  0.000000E+00       2691.17  0.000000E+00       2382.55       8354.27  0.000000E+00       13056.5       51.7542       6891.35 -1.000000E+10 -1.000000E+10 -1.000000E+10 -1.000000E+10 -1.000000E+10  0.000000E+00  0.000000E+00  0.000000E+00  0.000000E+00  0.000000E+00  0.000000E+00  0.000000E+00  0.000000E+00  0.000000E+00  0.000000E+00  0.000000E+00  0.000000E+00       Funtag_D
      37  3.922144E+05  3.509111E+05       41303.3       12.2507     0.0363315      0.622631      0.183096      0.240848    0.00100680 -1.229155E-04    0.00829595       1.04395       Opttag_D
      37       82.6173      0.949228       1.00000  0.000000E+00       1.00000  0.000000E+00      0.947191       98.5437       1.00000       33.4835  0.000000E+00      -1.30104  0.000000E+00      -1.30104  0.000000E+00  0.000000E+00       2.60209       83.1364     -0.105908        1       Scatag_D
      37       1940.69       5951.44  0.000000E+00  0.000000E+00       2690.34  0.000000E+00       2382.23       8348.28  0.000000E+00       13058.7       51.6989       6879.95 -1.000000E+10 -1.000000E+10 -1.000000E+10 -1.000000E+10 -1.000000E+10  0.000000E+00  0.000000E+00  0.000000E+00  0.000000E+00  0.000000E+00  0.000000E+00  0.000000E+00  0.000000E+00  0.000000E+00  0.000000E+00  0.000000E+00  0.000000E+00       Funtag_D
      38  3.921905E+05  3.508968E+05       41293.6       12.2874     0.0380513      0.668100      0.183083      0.240835    0.00104694 -1.368009E-04    0.00829862       1.04424       Opttag_D
      38       82.6139      0.949007       1.00000  0.000000E+00       1.00000  0.000000E+00      0.947212       98.5198       1.00000       33.4530  0.000000E+00      -1.30018  0.000000E+00      -1.30018  0.000000E+00  0.000000E+00       2.60037       83.1364     -0.105908        1       Scatag_D
      38       1941.94       5954.68  0.000000E+00  0.000000E+00       2689.37  0.000000E+00       2382.13       8344.53  0.000000E+00       13061.1       51.6810       6868.18 -1.000000E+10 -1.000000E+10 -1.000000E+10 -1.000000E+10 -1.000000E+10  0.000000E+00  0.000000E+00  0.000000E+00  0.000000E+00  0.000000E+00  0.000000E+00  0.000000E+00  0.000000E+00  0.000000E+00  0.000000E+00  0.000000E+00  0.000000E+00       Funtag_D
      39  3.921698E+05  3.508893E+05       41280.6       10.2921     0.0389345      0.691323      0.183077      0.240825    0.00106819 -1.438039E-04    0.00830011       1.04423       Opttag_D
      39       82.6124      0.948900       1.00000  0.000000E+00       1.00000  0.000000E+00      0.947224       98.5092       1.00000       33.4379  0.000000E+00      -1.29962  0.000000E+00      -1.29962  0.000000E+00  0.000000E+00       2.59924       83.1364     -0.105908        1       Scatag_D
      39       1942.63       5954.64  0.000000E+00  0.000000E+00       2688.94  0.000000E+00       2382.15       8336.12  0.000000E+00       13062.4       51.7047       6861.96 -1.000000E+10 -1.000000E+10 -1.000000E+10 -1.000000E+10 -1.000000E+10  0.000000E+00  0.000000E+00  0.000000E+00  0.000000E+00  0.000000E+00  0.000000E+00  0.000000E+00  0.000000E+00  0.000000E+00  0.000000E+00  0.000000E+00  0.000000E+00       Funtag_D
      40  3.921456E+05  3.508771E+05       41268.5       10.2345     0.0404389      0.730865      0.183065      0.240810    0.00110234 -1.543085E-04    0.00830313       1.04447       Opttag_D
      40       82.6100      0.948730       1.00000  0.000000E+00       1.00000  0.000000E+00      0.947245       98.4921       1.00000       33.4136  0.000000E+00      -1.29865  0.000000E+00      -1.29865  0.000000E+00  0.000000E+00       2.59731       83.1364     -0.105908        1       Scatag_D
      40       1944.05       5957.34  0.000000E+00  0.000000E+00       2688.29  0.000000E+00       2382.56       8328.67  0.000000E+00       13064.5       51.7986       6851.28 -1.000000E+10 -1.000000E+10 -1.000000E+10 -1.000000E+10 -1.000000E+10  0.000000E+00  0.000000E+00  0.000000E+00  0.000000E+00  0.000000E+00  0.000000E+00  0.000000E+00  0.000000E+00  0.000000E+00  0.000000E+00  0.000000E+00  0.000000E+00       Funtag_D
      41  3.921299E+05  3.508634E+05       41266.6       14.2540     0.0422752      0.779642      0.183052      0.240787    0.00114116 -1.725405E-04    0.00830957       1.04496       Opttag_D
      41       82.6072      0.948544       1.00000  0.000000E+00       1.00000  0.000000E+00      0.947267       98.4733       1.00000       33.3868  0.000000E+00      -1.29753  0.000000E+00      -1.29753  0.000000E+00  0.000000E+00       2.59506       83.1364     -0.105908        1       Scatag_D
      41       1947.06       5962.97  0.000000E+00  0.000000E+00       2687.48  0.000000E+00       2383.23       8329.17  0.000000E+00       13066.9       51.8954       6837.90 -1.000000E+10 -1.000000E+10 -1.000000E+10 -1.000000E+10 -1.000000E+10  0.000000E+00  0.000000E+00  0.000000E+00  0.000000E+00  0.000000E+00  0.000000E+00  0.000000E+00  0.000000E+00  0.000000E+00  0.000000E+00  0.000000E+00  0.000000E+00       Funtag_D
      42  3.921099E+05  3.508589E+05       41251.0       10.2129     0.0428377      0.795435      0.183047      0.240780    0.00115376 -1.797851E-04    0.00830911       1.04481       Opttag_D
      42       82.6063      0.948477       1.00000  0.000000E+00       1.00000  0.000000E+00      0.947274       98.4682       1.00000       33.3792  0.000000E+00      -1.29721  0.000000E+00      -1.29721  0.000000E+00  0.000000E+00       2.59443       83.1364     -0.105908        1       Scatag_D
      42       1946.85       5961.23  0.000000E+00  0.000000E+00       2687.29  0.000000E+00       2383.51       8320.29  0.000000E+00       13067.5       51.9298       6832.42 -1.000000E+10 -1.000000E+10 -1.000000E+10 -1.000000E+10 -1.000000E+10  0.000000E+00  0.000000E+00  0.000000E+00  0.000000E+00  0.000000E+00  0.000000E+00  0.000000E+00  0.000000E+00  0.000000E+00  0.000000E+00  0.000000E+00  0.000000E+00       Funtag_D
      43  3.920841E+05  3.508491E+05       41235.0       9.94467     0.0441745      0.833273      0.183039      0.240760    0.00118131 -2.010357E-04    0.00831219       1.04498       Opttag_D
      43       82.6043      0.948332       1.00000  0.000000E+00       1.00000  0.000000E+00      0.947291       98.4559       1.00000       33.3621  0.000000E+00      -1.29653  0.000000E+00      -1.29653  0.000000E+00  0.000000E+00       2.59305       83.1364     -0.105908        1       Scatag_D
      43       1948.29       5963.15  0.000000E+00  0.000000E+00       2686.79  0.000000E+00       2384.22       8311.67  0.000000E+00       13069.2       51.9823       6819.70 -1.000000E+10 -1.000000E+10 -1.000000E+10 -1.000000E+10 -1.000000E+10  0.000000E+00  0.000000E+00  0.000000E+00  0.000000E+00  0.000000E+00  0.000000E+00  0.000000E+00  0.000000E+00  0.000000E+00  0.000000E+00  0.000000E+00  0.000000E+00       Funtag_D
      44  3.920628E+05  3.508392E+05       41223.6       11.4701     0.0457257      0.877318      0.183030      0.240739    0.00120905 -2.356887E-04    0.00831574       1.04525       Opttag_D
      44       82.6021      0.948214       1.00000  0.000000E+00       1.00000  0.000000E+00      0.947311       98.4422       1.00000       33.3433  0.000000E+00      -1.29557  0.000000E+00      -1.29557  0.000000E+00  0.000000E+00       2.59115       83.1364     -0.105908        1       Scatag_D
      44       1949.96       5966.21  0.000000E+00  0.000000E+00       2686.31  0.000000E+00       2384.99       8307.29  0.000000E+00       13071.3       51.9911       6805.47 -1.000000E+10 -1.000000E+10 -1.000000E+10 -1.000000E+10 -1.000000E+10  0.000000E+00  0.000000E+00  0.000000E+00  0.000000E+00  0.000000E+00  0.000000E+00  0.000000E+00  0.000000E+00  0.000000E+00  0.000000E+00  0.000000E+00  0.000000E+00       Funtag_D
      45  3.920315E+05  3.508214E+05       41210.1       14.9402     0.0496140      0.986824      0.183011      0.240691    0.00125916 -3.443566E-04    0.00832861       1.04611       Opttag_D
      45       82.5973      0.948060       1.00000  0.000000E+00       1.00000  0.000000E+00      0.947361       98.4095       1.00000       33.3021  0.000000E+00      -1.29306  0.000000E+00      -1.29306  0.000000E+00  0.000000E+00       2.58611       83.1364     -0.105908        1       Scatag_D
      45       1955.99       5976.02  0.000000E+00  0.000000E+00       2685.41  0.000000E+00       2387.63       8303.86  0.000000E+00       13078.0       52.0034       6771.16 -1.000000E+10 -1.000000E+10 -1.000000E+10 -1.000000E+10 -1.000000E+10  0.000000E+00  0.000000E+00  0.000000E+00  0.000000E+00  0.000000E+00  0.000000E+00  0.000000E+00  0.000000E+00  0.000000E+00  0.000000E+00  0.000000E+00  0.000000E+00       Funtag_D
      46  3.920024E+05  3.508157E+05       41186.7       11.6266     0.0507337       1.02184      0.183005      0.240685    0.00127522 -4.122137E-04    0.00832693       1.04609       Opttag_D
      46       82.5962      0.948049       1.00000  0.000000E+00       1.00000  0.000000E+00      0.947376       98.4035       1.00000       33.2910  0.000000E+00      -1.29216  0.000000E+00      -1.29216  0.000000E+00  0.000000E+00       2.58432       83.1364     -0.105908        1       Scatag_D
      46       1955.21       5975.86  0.000000E+00  0.000000E+00       2685.21  0.000000E+00       2388.33       8291.95  0.000000E+00       13079.4       51.9190       6758.86 -1.000000E+10 -1.000000E+10 -1.000000E+10 -1.000000E+10 -1.000000E+10  0.000000E+00  0.000000E+00  0.000000E+00  0.000000E+00  0.000000E+00  0.000000E+00  0.000000E+00  0.000000E+00  0.000000E+00  0.000000E+00  0.000000E+00  0.000000E+00       Funtag_D
      47  3.919782E+05  3.508098E+05       41168.4       9.98761     0.0512497       1.04172      0.182998      0.240688    0.00129178 -4.826064E-04    0.00832131       1.04611       Opttag_D
      47       82.5956      0.948001       1.00000  0.000000E+00       1.00000  0.000000E+00      0.947384       98.4030       1.00000       33.2853  0.000000E+00      -1.29169  0.000000E+00      -1.29169  0.000000E+00  0.000000E+00       2.58338       83.1364     -0.105908        1       Scatag_D
      47       1952.57       5976.11  0.000000E+00  0.000000E+00       2685.15  0.000000E+00       2388.42       8284.10  0.000000E+00       13079.3       51.8354       6750.87 -1.000000E+10 -1.000000E+10 -1.000000E+10 -1.000000E+10 -1.000000E+10  0.000000E+00  0.000000E+00  0.000000E+00  0.000000E+00  0.000000E+00  0.000000E+00  0.000000E+00  0.000000E+00  0.000000E+00  0.000000E+00  0.000000E+00  0.000000E+00       Funtag_D
      48  3.919510E+05  3.507997E+05       41151.3       10.9904     0.0525373       1.08668      0.182986      0.240694    0.00132004 -6.127664E-04    0.00831546       1.04626       Opttag_D
      48       82.5945      0.947949       1.00000  0.000000E+00       1.00000  0.000000E+00      0.947401       98.4005       1.00000       33.2732  0.000000E+00      -1.29070  0.000000E+00      -1.29070  0.000000E+00  0.000000E+00       2.58141       83.1364     -0.105908        1       Scatag_D
      48       1949.83       5977.76  0.000000E+00  0.000000E+00       2684.99  0.000000E+00       2389.18       8282.71  0.000000E+00       13080.3       51.8320       6734.66 -1.000000E+10 -1.000000E+10 -1.000000E+10 -1.000000E+10 -1.000000E+10  0.000000E+00  0.000000E+00  0.000000E+00  0.000000E+00  0.000000E+00  0.000000E+00  0.000000E+00  0.000000E+00  0.000000E+00  0.000000E+00  0.000000E+00  0.000000E+00       Funtag_D
      49  3.919276E+05  3.507841E+05       41143.5       17.3074     0.0554885       1.18382      0.182968      0.240708    0.00136415 -8.635478E-04    0.00831142       1.04675       Opttag_D
      49       82.5925      0.947936       1.00000  0.000000E+00       1.00000  0.000000E+00      0.947438       98.3919       1.00000       33.2505  0.000000E+00      -1.28871  0.000000E+00      -1.28871  0.000000E+00  0.000000E+00       2.57743       83.1364     -0.105908        1       Scatag_D
      49       1947.93       5983.38  0.000000E+00  0.000000E+00       2684.85  0.000000E+00       2391.85       8296.68  0.000000E+00       13085.0       51.9603       6701.84 -1.000000E+10 -1.000000E+10 -1.000000E+10 -1.000000E+10 -1.000000E+10  0.000000E+00  0.000000E+00  0.000000E+00  0.000000E+00  0.000000E+00  0.000000E+00  0.000000E+00  0.000000E+00  0.000000E+00  0.000000E+00  0.000000E+00  0.000000E+00       Funtag_D
      50  3.918966E+05  3.507800E+05       41116.6       11.6043     0.0561337       1.20699      0.182962      0.240713    0.00137553 -9.189685E-04    0.00830675       1.04672       Opttag_D
      50       82.5924      0.947942       1.00000  0.000000E+00       1.00000  0.000000E+00      0.947446       98.3918       1.00000       33.2461  0.000000E+00      -1.28839  0.000000E+00      -1.28839  0.000000E+00  0.000000E+00       2.57679       83.1364     -0.105908        1       Scatag_D
      50       1945.74       5983.07  0.000000E+00  0.000000E+00       2684.87  0.000000E+00       2392.28       8279.53  0.000000E+00       13085.6       52.0553       6693.46 -1.000000E+10 -1.000000E+10 -1.000000E+10 -1.000000E+10 -1.000000E+10  0.000000E+00  0.000000E+00  0.000000E+00  0.000000E+00  0.000000E+00  0.000000E+00  0.000000E+00  0.000000E+00  0.000000E+00  0.000000E+00  0.000000E+00  0.000000E+00       Funtag_D
      51  3.918690E+05  3.507747E+05       41094.3       10.0847     0.0574459       1.25173      0.182954      0.240719    0.00139047   -0.00100602    0.00830318       1.04686       Opttag_D
      51       82.5923      0.947997       1.00000  0.000000E+00       1.00000  0.000000E+00      0.947462       98.3893       1.00000       33.2380  0.000000E+00      -1.28775  0.000000E+00      -1.28775  0.000000E+00  0.000000E+00       2.57549       83.1364     -0.105908        1       Scatag_D
      51       1944.07       5984.68  0.000000E+00  0.000000E+00       2684.87  0.000000E+00       2393.25       8270.34  0.000000E+00       13087.4       52.2227       6677.39 -1.000000E+10 -1.000000E+10 -1.000000E+10 -1.000000E+10 -1.000000E+10  0.000000E+00  0.000000E+00  0.000000E+00  0.000000E+00  0.000000E+00  0.000000E+00  0.000000E+00  0.000000E+00  0.000000E+00  0.000000E+00  0.000000E+00  0.000000E+00       Funtag_D
      52  3.918432E+05  3.507690E+05       41074.2       10.6279     0.0592752       1.31482      0.182943      0.240726    0.00140653   -0.00111843    0.00830078       1.04703       Opttag_D
      52       82.5924      0.948114       1.00000  0.000000E+00       1.00000  0.000000E+00      0.947486       98.3849       1.00000       33.2275  0.000000E+00      -1.28673  0.000000E+00      -1.28673  0.000000E+00  0.000000E+00       2.57347       83.1364     -0.105908        1       Scatag_D
      52       1942.95       5986.61  0.000000E+00  0.000000E+00       2684.97  0.000000E+00       2394.68       8267.16  0.000000E+00       13090.5       52.3298       6655.06 -1.000000E+10 -1.000000E+10 -1.000000E+10 -1.000000E+10 -1.000000E+10  0.000000E+00  0.000000E+00  0.000000E+00  0.000000E+00  0.000000E+00  0.000000E+00  0.000000E+00  0.000000E+00  0.000000E+00  0.000000E+00  0.000000E+00  0.000000E+00       Funtag_D
      53  3.918247E+05  3.507603E+05       41064.3       20.5244     0.0630659       1.44894      0.182926      0.240737    0.00143092   -0.00130534    0.00830170       1.04764       Opttag_D
      53       82.5941      0.948438       1.00000  0.000000E+00       1.00000  0.000000E+00      0.947540       98.3746       1.00000       33.2093  0.000000E+00      -1.28476  0.000000E+00      -1.28476  0.000000E+00  0.000000E+00       2.56951       83.1364     -0.105908        1       Scatag_D
      53       1943.38       5993.51  0.000000E+00  0.000000E+00       2685.47  0.000000E+00       2397.29       8286.63  0.000000E+00       13096.8       52.4829       6608.78 -1.000000E+10 -1.000000E+10 -1.000000E+10 -1.000000E+10 -1.000000E+10  0.000000E+00  0.000000E+00  0.000000E+00  0.000000E+00  0.000000E+00  0.000000E+00  0.000000E+00  0.000000E+00  0.000000E+00  0.000000E+00  0.000000E+00  0.000000E+00       Funtag_D
      54  3.917841E+05  3.507588E+05       41025.3       11.4807     0.0633100       1.46084      0.182922      0.240742    0.00143529   -0.00130414    0.00829868       1.04720       Opttag_D
      54       82.5945      0.948474       1.00000  0.000000E+00       1.00000  0.000000E+00      0.947545       98.3752       1.00000       33.2070  0.000000E+00      -1.28462  0.000000E+00      -1.28462  0.000000E+00  0.000000E+00       2.56924       83.1364     -0.105908        1       Scatag_D
      54       1941.96       5988.52  0.000000E+00  0.000000E+00       2685.51  0.000000E+00       2396.90       8259.56  0.000000E+00       13096.6       52.4068       6603.84 -1.000000E+10 -1.000000E+10 -1.000000E+10 -1.000000E+10 -1.000000E+10  0.000000E+00  0.000000E+00  0.000000E+00  0.000000E+00  0.000000E+00  0.000000E+00  0.000000E+00  0.000000E+00  0.000000E+00  0.000000E+00  0.000000E+00  0.000000E+00       Funtag_D
      55  3.917611E+05  3.507553E+05       41005.8       10.4176     0.0633524       1.46881      0.182917      0.240744    0.00144505   -0.00128458    0.00829623       1.04693       Opttag_D
      55       82.5951      0.948466       1.00000  0.000000E+00       1.00000  0.000000E+00      0.947549       98.3770       1.00000       33.2049  0.000000E+00      -1.28468  0.000000E+00      -1.28468  0.000000E+00  0.000000E+00       2.56935       83.1364     -0.105908        1       Scatag_D
      55       1940.82       5985.46  0.000000E+00  0.000000E+00       2685.55  0.000000E+00       2395.88       8250.73  0.000000E+00       13095.2       52.3020       6599.82 -1.000000E+10 -1.000000E+10 -1.000000E+10 -1.000000E+10 -1.000000E+10  0.000000E+00  0.000000E+00  0.000000E+00  0.000000E+00  0.000000E+00  0.000000E+00  0.000000E+00  0.000000E+00  0.000000E+00  0.000000E+00  0.000000E+00  0.000000E+00       Funtag_D

 An atom has moved more than     1.50 A from the initial/last
 position - if this was an accepted trial point, probably need to update the
 non-bonded contact list.

      56  3.917217E+05  3.507460E+05       40975.7       12.6978     0.0649191       1.53777      0.182901      0.240752    0.00147117   -0.00130836    0.00829583       1.04660       Opttag_D
      56       82.5973      0.948586       1.00000  0.000000E+00       1.00000  0.000000E+00      0.947577       98.3786       1.00000       33.1954  0.000000E+00      -1.28401  0.000000E+00      -1.28401  0.000000E+00  0.000000E+00       2.56802       83.1364     -0.105908        1       Scatag_D
      56       1940.63       5981.71  0.000000E+00  0.000000E+00       2685.93  0.000000E+00       2395.24       8249.68  0.000000E+00       13095.4       52.2502       6574.94 -1.000000E+10 -1.000000E+10 -1.000000E+10 -1.000000E+10 -1.000000E+10  0.000000E+00  0.000000E+00  0.000000E+00  0.000000E+00  0.000000E+00  0.000000E+00  0.000000E+00  0.000000E+00  0.000000E+00  0.000000E+00  0.000000E+00  0.000000E+00       Funtag_D

 An atom has moved more than     1.50 A from the initial/last
 position - need to update the non-bonded contact list.


 ############################################################################################################
 ###################################################### setting up contact terms
 ############################################################################################################

      57  3.916969E+05  3.507288E+05       40968.1       27.5708     0.0690680       1.71405      0.182871      0.240784    0.00151975   -0.00138467    0.00830209       1.04685       Opttag_D
      57       82.6038      0.948957       1.00000  0.000000E+00       1.00000  0.000000E+00      0.947644       98.3848       1.00000       33.1795  0.000000E+00      -1.28262  0.000000E+00      -1.28262  0.000000E+00  0.000000E+00       2.56524       83.1364     -0.105908        1       Scatag_D
      57       1943.56       5984.51  0.000000E+00  0.000000E+00       2687.16  0.000000E+00       2395.39       8285.42  0.000000E+00       13099.0       52.3861       6520.67 -1.000000E+10 -1.000000E+10 -1.000000E+10 -1.000000E+10 -1.000000E+10  0.000000E+00  0.000000E+00  0.000000E+00  0.000000E+00  0.000000E+00  0.000000E+00  0.000000E+00  0.000000E+00  0.000000E+00  0.000000E+00  0.000000E+00  0.000000E+00       Funtag_D
      58  3.916354E+05  3.507234E+05       40912.1       14.0207     0.0688747       1.71218      0.182867      0.240782    0.00153490   -0.00135581    0.00829466       1.04613       Opttag_D
      58       82.6039      0.948847       1.00000  0.000000E+00       1.00000  0.000000E+00      0.947642       98.3883       1.00000       33.1783  0.000000E+00      -1.28284  0.000000E+00      -1.28284  0.000000E+00  0.000000E+00       2.56568       83.1364     -0.105908        1       Scatag_D
      58       1940.08       5976.36  0.000000E+00  0.000000E+00       2687.20  0.000000E+00       2394.45       8245.46  0.000000E+00       13096.8       52.4067       6519.35 -1.000000E+10 -1.000000E+10 -1.000000E+10 -1.000000E+10 -1.000000E+10  0.000000E+00  0.000000E+00  0.000000E+00  0.000000E+00  0.000000E+00  0.000000E+00  0.000000E+00  0.000000E+00  0.000000E+00  0.000000E+00  0.000000E+00  0.000000E+00       Funtag_D
      59  3.916084E+05  3.507177E+05       40890.7       10.7446     0.0690812       1.72406      0.182864      0.240782    0.00155075   -0.00134410    0.00829037       1.04603       Opttag_D
      59       82.6044      0.948785       1.00000  0.000000E+00       1.00000  0.000000E+00      0.947643       98.3929       1.00000       33.1770  0.000000E+00      -1.28304  0.000000E+00      -1.28304  0.000000E+00  0.000000E+00       2.56608       83.1364     -0.105908        1       Scatag_D
      59       1938.08       5975.20  0.000000E+00  0.000000E+00       2687.33  0.000000E+00       2393.83       8233.40  0.000000E+00       13095.6       52.5074       6514.72 -1.000000E+10 -1.000000E+10 -1.000000E+10 -1.000000E+10 -1.000000E+10  0.000000E+00  0.000000E+00  0.000000E+00  0.000000E+00  0.000000E+00  0.000000E+00  0.000000E+00  0.000000E+00  0.000000E+00  0.000000E+00  0.000000E+00  0.000000E+00       Funtag_D
      60  3.915787E+05  3.507062E+05       40872.5       10.9623     0.0702979       1.77837      0.182852      0.240792    0.00158307   -0.00133758    0.00828822       1.04601       Opttag_D
      60       82.6069      0.948781       1.00000  0.000000E+00       1.00000  0.000000E+00      0.947661       98.4009       1.00000       33.1728  0.000000E+00      -1.28314  0.000000E+00      -1.28314  0.000000E+00  0.000000E+00       2.56628       83.1364     -0.105908        1       Scatag_D
      60       1937.07       5974.92  0.000000E+00  0.000000E+00       2687.89  0.000000E+00       2393.00       8228.17  0.000000E+00       13095.7       52.7125       6502.97 -1.000000E+10 -1.000000E+10 -1.000000E+10 -1.000000E+10 -1.000000E+10  0.000000E+00  0.000000E+00  0.000000E+00  0.000000E+00  0.000000E+00  0.000000E+00  0.000000E+00  0.000000E+00  0.000000E+00  0.000000E+00  0.000000E+00  0.000000E+00       Funtag_D
      61  3.915569E+05  3.506883E+05       40868.6       17.8385     0.0700658       1.76751      0.182838      0.240782    0.00163348   -0.00125028    0.00828536       1.04617       Opttag_D
      61       82.6079      0.948570       1.00000  0.000000E+00       1.00000  0.000000E+00      0.947656       98.4157       1.00000       33.1683  0.000000E+00      -1.28441  0.000000E+00      -1.28441  0.000000E+00  0.000000E+00       2.56882       83.1364     -0.105908        1       Scatag_D
      61       1935.73       5976.79  0.000000E+00  0.000000E+00       2688.26  0.000000E+00       2389.54       8237.00  0.000000E+00       13088.6       52.8030       6499.91 -1.000000E+10 -1.000000E+10 -1.000000E+10 -1.000000E+10 -1.000000E+10  0.000000E+00  0.000000E+00  0.000000E+00  0.000000E+00  0.000000E+00  0.000000E+00  0.000000E+00  0.000000E+00  0.000000E+00  0.000000E+00  0.000000E+00  0.000000E+00       Funtag_D
      62  3.915208E+05  3.506783E+05       40842.5       10.6830     0.0714166       1.82041      0.182825      0.240796    0.00166153   -0.00124920    0.00828403       1.04576       Opttag_D
      62       82.6113      0.948627       1.00000  0.000000E+00       1.00000  0.000000E+00      0.947680       98.4216       1.00000       33.1636  0.000000E+00      -1.28435  0.000000E+00      -1.28435  0.000000E+00  0.000000E+00       2.56869       83.1364     -0.105908        1       Scatag_D
      62       1935.11       5972.09  0.000000E+00  0.000000E+00       2688.96  0.000000E+00       2388.67       8220.33  0.000000E+00       13089.1       52.8293       6495.41 -1.000000E+10 -1.000000E+10 -1.000000E+10 -1.000000E+10 -1.000000E+10  0.000000E+00  0.000000E+00  0.000000E+00  0.000000E+00  0.000000E+00  0.000000E+00  0.000000E+00  0.000000E+00  0.000000E+00  0.000000E+00  0.000000E+00  0.000000E+00       Funtag_D
      63  3.915038E+05  3.506734E+05       40830.4       8.41810     0.0716921       1.82723      0.182820      0.240799    0.00167546   -0.00123761    0.00828460       1.04555       Opttag_D
      63       82.6125      0.948630       1.00000  0.000000E+00       1.00000  0.000000E+00      0.947687       98.4253       1.00000       33.1609  0.000000E+00      -1.28448  0.000000E+00      -1.28448  0.000000E+00  0.000000E+00       2.56896       83.1364     -0.105908        1       Scatag_D
      63       1935.38       5969.71  0.000000E+00  0.000000E+00       2689.21  0.000000E+00       2387.92       8212.53  0.000000E+00       13088.0       52.7396       6494.98 -1.000000E+10 -1.000000E+10 -1.000000E+10 -1.000000E+10 -1.000000E+10  0.000000E+00  0.000000E+00  0.000000E+00  0.000000E+00  0.000000E+00  0.000000E+00  0.000000E+00  0.000000E+00  0.000000E+00  0.000000E+00  0.000000E+00  0.000000E+00       Funtag_D
      64  3.914814E+05  3.506604E+05       40821.0       9.26587     0.0720928       1.83376      0.182808      0.240799    0.00171194   -0.00119438    0.00828691       1.04532       Opttag_D
      64       82.6151      0.948593       1.00000  0.000000E+00       1.00000  0.000000E+00      0.947697       98.4360       1.00000       33.1552  0.000000E+00      -1.28492  0.000000E+00      -1.28492  0.000000E+00  0.000000E+00       2.56984       83.1364     -0.105908        1       Scatag_D
      64       1936.46       5967.08  0.000000E+00  0.000000E+00       2689.77  0.000000E+00       2386.55       8208.52  0.000000E+00       13084.3       52.6018       6495.75 -1.000000E+10 -1.000000E+10 -1.000000E+10 -1.000000E+10 -1.000000E+10  0.000000E+00  0.000000E+00  0.000000E+00  0.000000E+00  0.000000E+00  0.000000E+00  0.000000E+00  0.000000E+00  0.000000E+00  0.000000E+00  0.000000E+00  0.000000E+00       Funtag_D
      65  3.914587E+05  3.506372E+05       40821.5       12.8531     0.0728273       1.84333      0.182788      0.240799    0.00177719   -0.00111505    0.00828960       1.04499       Opttag_D
      65       82.6201      0.948553       1.00000  0.000000E+00       1.00000  0.000000E+00      0.947713       98.4587       1.00000       33.1450  0.000000E+00      -1.28569  0.000000E+00      -1.28569  0.000000E+00  0.000000E+00       2.57138       83.1364     -0.105908        1       Scatag_D
      65       1937.72       5963.34  0.000000E+00  0.000000E+00       2690.87  0.000000E+00       2385.23       8213.78  0.000000E+00       13077.4       52.4427       6500.70 -1.000000E+10 -1.000000E+10 -1.000000E+10 -1.000000E+10 -1.000000E+10  0.000000E+00  0.000000E+00  0.000000E+00  0.000000E+00  0.000000E+00  0.000000E+00  0.000000E+00  0.000000E+00  0.000000E+00  0.000000E+00  0.000000E+00  0.000000E+00       Funtag_D
      66  3.914320E+05  3.506206E+05       40811.4       10.4349     0.0730850       1.83693      0.182773      0.240794    0.00182405   -0.00105504    0.00828844       1.04484       Opttag_D
      66       82.6234      0.948525       1.00000  0.000000E+00       1.00000  0.000000E+00      0.947718       98.4778       1.00000       33.1383  0.000000E+00      -1.28635  0.000000E+00      -1.28635  0.000000E+00  0.000000E+00       2.57270       83.1364     -0.105908        1       Scatag_D
      66       1937.17       5961.56  0.000000E+00  0.000000E+00       2691.62  0.000000E+00       2384.98       8207.88  0.000000E+00       13071.8       52.3844       6504.04 -1.000000E+10 -1.000000E+10 -1.000000E+10 -1.000000E+10 -1.000000E+10  0.000000E+00  0.000000E+00  0.000000E+00  0.000000E+00  0.000000E+00  0.000000E+00  0.000000E+00  0.000000E+00  0.000000E+00  0.000000E+00  0.000000E+00  0.000000E+00       Funtag_D
      67  3.914081E+05  3.506055E+05       40802.6       10.2453     0.0730738       1.82043      0.182762      0.240792    0.00186643   -0.00102450    0.00828559       1.04478       Opttag_D
      67       82.6255      0.948455       1.00000  0.000000E+00       1.00000  0.000000E+00      0.947715       98.4959       1.00000       33.1327  0.000000E+00      -1.28703  0.000000E+00      -1.28703  0.000000E+00  0.000000E+00       2.57406       83.1364     -0.105908        1       Scatag_D
      67       1935.84       5960.95  0.000000E+00  0.000000E+00       2692.15  0.000000E+00       2384.89       8205.13  0.000000E+00       13066.6       52.4073       6504.67 -1.000000E+10 -1.000000E+10 -1.000000E+10 -1.000000E+10 -1.000000E+10  0.000000E+00  0.000000E+00  0.000000E+00  0.000000E+00  0.000000E+00  0.000000E+00  0.000000E+00  0.000000E+00  0.000000E+00  0.000000E+00  0.000000E+00  0.000000E+00       Funtag_D
      68  3.913783E+05  3.505796E+05       40798.7       10.9910     0.0731275       1.79136      0.182742      0.240794    0.00193930 -9.801848E-04    0.00828598       1.04478       Opttag_D
      68       82.6296      0.948377       1.00000  0.000000E+00       1.00000  0.000000E+00      0.947708       98.5306       1.00000       33.1233  0.000000E+00      -1.28848  0.000000E+00      -1.28848  0.000000E+00  0.000000E+00       2.57697       83.1364     -0.105908        1       Scatag_D
      68       1936.02       5960.88  0.000000E+00  0.000000E+00       2693.19  0.000000E+00       2384.77       8206.40  0.000000E+00       13058.1       52.4993       6506.90 -1.000000E+10 -1.000000E+10 -1.000000E+10 -1.000000E+10 -1.000000E+10  0.000000E+00  0.000000E+00  0.000000E+00  0.000000E+00  0.000000E+00  0.000000E+00  0.000000E+00  0.000000E+00  0.000000E+00  0.000000E+00  0.000000E+00  0.000000E+00       Funtag_D
      69  3.913663E+05  3.505513E+05       40815.0       16.5470     0.0731202       1.75281      0.182719      0.240798    0.00201888 -9.475844E-04    0.00829029       1.04482       Opttag_D
      69       82.6340      0.948314       1.00000  0.000000E+00       1.00000  0.000000E+00      0.947696       98.5726       1.00000       33.1142  0.000000E+00      -1.29056  0.000000E+00      -1.29056  0.000000E+00  0.000000E+00       2.58111       83.1364     -0.105908        1       Scatag_D
      69       1938.04       5961.35  0.000000E+00  0.000000E+00       2694.46  0.000000E+00       2384.05       8225.56  0.000000E+00       13050.2       52.6179       6508.78 -1.000000E+10 -1.000000E+10 -1.000000E+10 -1.000000E+10 -1.000000E+10  0.000000E+00  0.000000E+00  0.000000E+00  0.000000E+00  0.000000E+00  0.000000E+00  0.000000E+00  0.000000E+00  0.000000E+00  0.000000E+00  0.000000E+00  0.000000E+00       Funtag_D
      70  3.913398E+05  3.505508E+05       40789.0       8.61336     0.0731683       1.75620      0.182719      0.240798    0.00202020 -9.685935E-04    0.00828771       1.04466       Opttag_D
      70       82.6337      0.948264       1.00000  0.000000E+00       1.00000  0.000000E+00      0.947697       98.5702       1.00000       33.1142  0.000000E+00      -1.29075  0.000000E+00      -1.29075  0.000000E+00  0.000000E+00       2.58150       83.1364     -0.105908        1       Scatag_D
      70       1936.83       5959.53  0.000000E+00  0.000000E+00       2694.44  0.000000E+00       2383.59       8205.14  0.000000E+00       13050.4       52.5758       6506.49 -1.000000E+10 -1.000000E+10 -1.000000E+10 -1.000000E+10 -1.000000E+10  0.000000E+00  0.000000E+00  0.000000E+00  0.000000E+00  0.000000E+00  0.000000E+00  0.000000E+00  0.000000E+00  0.000000E+00  0.000000E+00  0.000000E+00  0.000000E+00       Funtag_D
      71  3.913227E+05  3.505448E+05       40777.9       7.29472     0.0732543       1.75366      0.182715      0.240798    0.00203728 -9.953983E-04    0.00828609       1.04453       Opttag_D
      71       82.6340      0.948188       1.00000  0.000000E+00       1.00000  0.000000E+00      0.947697       98.5756       1.00000       33.1126  0.000000E+00      -1.29156  0.000000E+00      -1.29156  0.000000E+00  0.000000E+00       2.58313       83.1364     -0.105908        1       Scatag_D
      71       1936.07       5958.02  0.000000E+00  0.000000E+00       2694.71  0.000000E+00       2382.84       8200.60  0.000000E+00       13049.4       52.5189       6503.78 -1.000000E+10 -1.000000E+10 -1.000000E+10 -1.000000E+10 -1.000000E+10  0.000000E+00  0.000000E+00  0.000000E+00  0.000000E+00  0.000000E+00  0.000000E+00  0.000000E+00  0.000000E+00  0.000000E+00  0.000000E+00  0.000000E+00  0.000000E+00       Funtag_D
      72  3.913055E+05  3.505316E+05       40773.9       8.37613     0.0733633       1.74155      0.182706      0.240796    0.00207427   -0.00102438    0.00828530       1.04447       Opttag_D
      72       82.6352      0.948081       1.00000  0.000000E+00       1.00000  0.000000E+00      0.947692       98.5913       1.00000       33.1089  0.000000E+00      -1.29319  0.000000E+00      -1.29319  0.000000E+00  0.000000E+00       2.58638       83.1364     -0.105908        1       Scatag_D
      72       1935.71       5957.37  0.000000E+00  0.000000E+00       2695.37  0.000000E+00       2382.14       8202.34  0.000000E+00       13047.0       52.4404       6501.57 -1.000000E+10 -1.000000E+10 -1.000000E+10 -1.000000E+10 -1.000000E+10  0.000000E+00  0.000000E+00  0.000000E+00  0.000000E+00  0.000000E+00  0.000000E+00  0.000000E+00  0.000000E+00  0.000000E+00  0.000000E+00  0.000000E+00  0.000000E+00       Funtag_D
      73  3.912855E+05  3.505058E+05       40779.7       13.2195     0.0735927       1.72924      0.182689      0.240795    0.00214684   -0.00110141    0.00828411       1.04444       Opttag_D
      73       82.6371      0.947854       1.00000  0.000000E+00       1.00000  0.000000E+00      0.947680       98.6243       1.00000       33.1018  0.000000E+00      -1.29668  0.000000E+00      -1.29668  0.000000E+00  0.000000E+00       2.59336       83.1364     -0.105908        1       Scatag_D
      73       1935.15       5957.04  0.000000E+00  0.000000E+00       2696.73  0.000000E+00       2381.87       8212.76  0.000000E+00       13043.5       52.2324       6500.41 -1.000000E+10 -1.000000E+10 -1.000000E+10 -1.000000E+10 -1.000000E+10  0.000000E+00  0.000000E+00  0.000000E+00  0.000000E+00  0.000000E+00  0.000000E+00  0.000000E+00  0.000000E+00  0.000000E+00  0.000000E+00  0.000000E+00  0.000000E+00       Funtag_D
      74  3.912630E+05  3.504877E+05       40775.3       8.04625     0.0737876       1.76494      0.182677      0.240788    0.00219786   -0.00113558    0.00828353       1.04450       Opttag_D
      74       82.6387      0.947763       1.00000  0.000000E+00       1.00000  0.000000E+00      0.947667       98.6526       1.00000       33.0969  0.000000E+00      -1.29942  0.000000E+00      -1.29942  0.000000E+00  0.000000E+00       2.59885       83.1364     -0.105908        1       Scatag_D
      74       1934.88       5957.67  0.000000E+00  0.000000E+00       2697.88  0.000000E+00       2382.26       8206.66  0.000000E+00       13041.4       52.0913       6502.54 -1.000000E+10 -1.000000E+10 -1.000000E+10 -1.000000E+10 -1.000000E+10  0.000000E+00  0.000000E+00  0.000000E+00  0.000000E+00  0.000000E+00  0.000000E+00  0.000000E+00  0.000000E+00  0.000000E+00  0.000000E+00  0.000000E+00  0.000000E+00       Funtag_D
      75  3.912518E+05  3.504813E+05       40770.5       6.12334     0.0738253       1.77427      0.182674      0.240786    0.00221585   -0.00114512    0.00828137       1.04444       Opttag_D
      75       82.6389      0.947705       1.00000  0.000000E+00       1.00000  0.000000E+00      0.947661       98.6620       1.00000       33.0950  0.000000E+00      -1.30024  0.000000E+00      -1.30024  0.000000E+00  0.000000E+00       2.60048       83.1364     -0.105908        1       Scatag_D
      75       1933.87       5957.03  0.000000E+00  0.000000E+00       2698.14  0.000000E+00       2382.14       8203.87  0.000000E+00       13040.9       52.0581       6502.51 -1.000000E+10 -1.000000E+10 -1.000000E+10 -1.000000E+10 -1.000000E+10  0.000000E+00  0.000000E+00  0.000000E+00  0.000000E+00  0.000000E+00  0.000000E+00  0.000000E+00  0.000000E+00  0.000000E+00  0.000000E+00  0.000000E+00  0.000000E+00       Funtag_D
      76  3.912377E+05  3.504666E+05       40771.1       6.56659     0.0739398       1.79570      0.182666      0.240785    0.00225701   -0.00116564    0.00827956       1.04436       Opttag_D
      76       82.6394      0.947594       1.00000  0.000000E+00       1.00000  0.000000E+00      0.947644       98.6848       1.00000       33.0909  0.000000E+00      -1.30213  0.000000E+00      -1.30213  0.000000E+00  0.000000E+00       2.60427       83.1364     -0.105908        1       Scatag_D
      76       1933.02       5956.13  0.000000E+00  0.000000E+00       2698.75  0.000000E+00       2381.87       8206.32  0.000000E+00       13039.8       52.0444       6503.11 -1.000000E+10 -1.000000E+10 -1.000000E+10 -1.000000E+10 -1.000000E+10  0.000000E+00  0.000000E+00  0.000000E+00  0.000000E+00  0.000000E+00  0.000000E+00  0.000000E+00  0.000000E+00  0.000000E+00  0.000000E+00  0.000000E+00  0.000000E+00       Funtag_D
      77  3.912312E+05  3.504466E+05       40784.5       12.0760     0.0741732       1.82791      0.182653      0.240781    0.00231322   -0.00116963    0.00827897       1.04434       Opttag_D
      77       82.6408      0.947507       1.00000  0.000000E+00       1.00000  0.000000E+00      0.947619       98.7210       1.00000       33.0852  0.000000E+00      -1.30492  0.000000E+00      -1.30492  0.000000E+00  0.000000E+00       2.60983       83.1364     -0.105908        1       Scatag_D
      77       1932.75       5955.88  0.000000E+00  0.000000E+00       2699.71  0.000000E+00       2381.61       8219.08  0.000000E+00       13038.1       52.0745       6505.34 -1.000000E+10 -1.000000E+10 -1.000000E+10 -1.000000E+10 -1.000000E+10  0.000000E+00  0.000000E+00  0.000000E+00  0.000000E+00  0.000000E+00  0.000000E+00  0.000000E+00  0.000000E+00  0.000000E+00  0.000000E+00  0.000000E+00  0.000000E+00       Funtag_D
      78  3.912182E+05  3.504439E+05       40774.3       6.85377     0.0742069       1.83048      0.182652      0.240783    0.00232094   -0.00116878    0.00827839       1.04431       Opttag_D
      78       82.6411      0.947497       1.00000  0.000000E+00       1.00000  0.000000E+00      0.947615       98.7258       1.00000       33.0844  0.000000E+00      -1.30520  0.000000E+00      -1.30520  0.000000E+00  0.000000E+00       2.61039       83.1364     -0.105908        1       Scatag_D
      78       1932.48       5955.55  0.000000E+00  0.000000E+00       2699.79  0.000000E+00       2381.48       8209.88  0.000000E+00       13037.8       52.0917       6505.19 -1.000000E+10 -1.000000E+10 -1.000000E+10 -1.000000E+10 -1.000000E+10  0.000000E+00  0.000000E+00  0.000000E+00  0.000000E+00  0.000000E+00  0.000000E+00  0.000000E+00  0.000000E+00  0.000000E+00  0.000000E+00  0.000000E+00  0.000000E+00       Funtag_D
      79  3.912078E+05  3.504350E+05       40772.8       5.51239     0.0743448       1.84312      0.182645      0.240784    0.00234605   -0.00116299    0.00827781       1.04430       Opttag_D
      79       82.6421      0.947484       1.00000  0.000000E+00       1.00000  0.000000E+00      0.947603       98.7436       1.00000       33.0820  0.000000E+00      -1.30638  0.000000E+00      -1.30638  0.000000E+00  0.000000E+00       2.61275       83.1364     -0.105908        1       Scatag_D
      79       1932.21       5955.47  0.000000E+00  0.000000E+00       2700.20  0.000000E+00       2381.25       8208.73  0.000000E+00       13037.0       52.1151       6505.82 -1.000000E+10 -1.000000E+10 -1.000000E+10 -1.000000E+10 -1.000000E+10  0.000000E+00  0.000000E+00  0.000000E+00  0.000000E+00  0.000000E+00  0.000000E+00  0.000000E+00  0.000000E+00  0.000000E+00  0.000000E+00  0.000000E+00  0.000000E+00       Funtag_D
      80  3.911994E+05  3.504232E+05       40776.1       5.96113     0.0745367       1.85954      0.182639      0.240783    0.00237908   -0.00115659    0.00827738       1.04433       Opttag_D
      80       82.6435      0.947471       1.00000  0.000000E+00       1.00000  0.000000E+00      0.947587       98.7679       1.00000       33.0788  0.000000E+00      -1.30794  0.000000E+00      -1.30794  0.000000E+00  0.000000E+00       2.61588       83.1364     -0.105908        1       Scatag_D
      80       1932.01       5955.75  0.000000E+00  0.000000E+00       2700.74  0.000000E+00       2381.16       8211.25  0.000000E+00       13036.2       52.1084       6506.90 -1.000000E+10 -1.000000E+10 -1.000000E+10 -1.000000E+10 -1.000000E+10  0.000000E+00  0.000000E+00  0.000000E+00  0.000000E+00  0.000000E+00  0.000000E+00  0.000000E+00  0.000000E+00  0.000000E+00  0.000000E+00  0.000000E+00  0.000000E+00       Funtag_D
      81  3.911858E+05  3.503975E+05       40788.3       9.50306     0.0749971       1.88894      0.182622      0.240785    0.00245154   -0.00116395    0.00827653       1.04440       Opttag_D
      81       82.6465      0.947424       1.00000  0.000000E+00       1.00000  0.000000E+00      0.947551       98.8233       1.00000       33.0721  0.000000E+00      -1.31106  0.000000E+00      -1.31106  0.000000E+00  0.000000E+00       2.62211       83.1364     -0.105908        1       Scatag_D
      81       1931.61       5956.51  0.000000E+00  0.000000E+00       2701.74  0.000000E+00       2381.22       8221.29  0.000000E+00       13035.8       51.9931       6508.13 -1.000000E+10 -1.000000E+10 -1.000000E+10 -1.000000E+10 -1.000000E+10  0.000000E+00  0.000000E+00  0.000000E+00  0.000000E+00  0.000000E+00  0.000000E+00  0.000000E+00  0.000000E+00  0.000000E+00  0.000000E+00  0.000000E+00  0.000000E+00       Funtag_D
      82  3.911744E+05  3.503753E+05       40799.1       7.95059     0.0754664       1.91357      0.182609      0.240787    0.00251405   -0.00116830    0.00827688       1.04454       Opttag_D
      82       82.6496      0.947441       1.00000  0.000000E+00       1.00000  0.000000E+00      0.947517       98.8767       1.00000       33.0667  0.000000E+00      -1.31378  0.000000E+00      -1.31378  0.000000E+00  0.000000E+00       2.62756       83.1364     -0.105908        1       Scatag_D
      82       1931.77       5958.11  0.000000E+00  0.000000E+00       2702.65  0.000000E+00       2381.58       8226.13  0.000000E+00       13037.0       51.8639       6510.09 -1.000000E+10 -1.000000E+10 -1.000000E+10 -1.000000E+10 -1.000000E+10  0.000000E+00  0.000000E+00  0.000000E+00  0.000000E+00  0.000000E+00  0.000000E+00  0.000000E+00  0.000000E+00  0.000000E+00  0.000000E+00  0.000000E+00  0.000000E+00       Funtag_D
      83  3.911671E+05  3.503754E+05       40791.7       5.52995     0.0754112       1.90592      0.182610      0.240789    0.00251364   -0.00117458    0.00827659       1.04450       Opttag_D
      83       82.6492      0.947392       1.00000  0.000000E+00       1.00000  0.000000E+00      0.947521       98.8723       1.00000       33.0667  0.000000E+00      -1.31325  0.000000E+00      -1.31325  0.000000E+00  0.000000E+00       2.62651       83.1364     -0.105908        1       Scatag_D
      83       1931.64       5957.66  0.000000E+00  0.000000E+00       2702.40  0.000000E+00       2381.41       8222.00  0.000000E+00       13036.5       51.8336       6508.24 -1.000000E+10 -1.000000E+10 -1.000000E+10 -1.000000E+10 -1.000000E+10  0.000000E+00  0.000000E+00  0.000000E+00  0.000000E+00  0.000000E+00  0.000000E+00  0.000000E+00  0.000000E+00  0.000000E+00  0.000000E+00  0.000000E+00  0.000000E+00       Funtag_D
      84  3.911590E+05  3.503690E+05       40790.0       5.20535     0.0754631       1.89881      0.182605      0.240794    0.00253163   -0.00119619    0.00827744       1.04450       Opttag_D
      84       82.6494      0.947318       1.00000  0.000000E+00       1.00000  0.000000E+00      0.947516       98.8813       1.00000       33.0652  0.000000E+00      -1.31311  0.000000E+00      -1.31311  0.000000E+00  0.000000E+00       2.62622       83.1364     -0.105908        1       Scatag_D
      84       1932.03       5957.71  0.000000E+00  0.000000E+00       2702.22  0.000000E+00       2381.39       8222.66  0.000000E+00       13036.1       51.7659       6506.10 -1.000000E+10 -1.000000E+10 -1.000000E+10 -1.000000E+10 -1.000000E+10  0.000000E+00  0.000000E+00  0.000000E+00  0.000000E+00  0.000000E+00  0.000000E+00  0.000000E+00  0.000000E+00  0.000000E+00  0.000000E+00  0.000000E+00  0.000000E+00       Funtag_D
      85  3.911511E+05  3.503559E+05       40795.2       6.20643     0.0756632       1.89670      0.182595      0.240803    0.00256853   -0.00122940    0.00827926       1.04456       Opttag_D
      85       82.6506      0.947254       1.00000  0.000000E+00       1.00000  0.000000E+00      0.947501       98.9071       1.00000       33.0625  0.000000E+00      -1.31367  0.000000E+00      -1.31367  0.000000E+00  0.000000E+00       2.62733       83.1364     -0.105908        1       Scatag_D
      85       1932.88       5958.41  0.000000E+00  0.000000E+00       2702.28  0.000000E+00       2381.65       8227.04  0.000000E+00       13036.0       51.6968       6505.19 -1.000000E+10 -1.000000E+10 -1.000000E+10 -1.000000E+10 -1.000000E+10  0.000000E+00  0.000000E+00  0.000000E+00  0.000000E+00  0.000000E+00  0.000000E+00  0.000000E+00  0.000000E+00  0.000000E+00  0.000000E+00  0.000000E+00  0.000000E+00       Funtag_D
      86  3.911401E+05  3.503309E+05       40809.2       7.07030     0.0761350       1.89811      0.182581      0.240820    0.00263896   -0.00129230    0.00828291       1.04471       Opttag_D
      86       82.6535      0.947218       1.00000  0.000000E+00       1.00000  0.000000E+00      0.947467       98.9639       1.00000       33.0575  0.000000E+00      -1.31521  0.000000E+00      -1.31521  0.000000E+00  0.000000E+00       2.63043       83.1364     -0.105908        1       Scatag_D
      86       1934.59       5960.14  0.000000E+00  0.000000E+00       2702.66  0.000000E+00       2382.30       8235.21  0.000000E+00       13036.5       51.6172       6506.22 -1.000000E+10 -1.000000E+10 -1.000000E+10 -1.000000E+10 -1.000000E+10  0.000000E+00  0.000000E+00  0.000000E+00  0.000000E+00  0.000000E+00  0.000000E+00  0.000000E+00  0.000000E+00  0.000000E+00  0.000000E+00  0.000000E+00  0.000000E+00       Funtag_D
      87  3.911302E+05  3.503160E+05       40814.2       5.92399     0.0764652       1.90054      0.182574      0.240830    0.00268063   -0.00133094    0.00828351       1.04481       Opttag_D
      87       82.6557      0.947247       1.00000  0.000000E+00       1.00000  0.000000E+00      0.947442       99.0025       1.00000       33.0549  0.000000E+00      -1.31631  0.000000E+00      -1.31631  0.000000E+00  0.000000E+00       2.63262       83.1364     -0.105908        1       Scatag_D
      87       1934.87       5961.20  0.000000E+00  0.000000E+00       2703.01  0.000000E+00       2382.57       8236.41  0.000000E+00       13037.0       51.6155       6507.50 -1.000000E+10 -1.000000E+10 -1.000000E+10 -1.000000E+10 -1.000000E+10  0.000000E+00  0.000000E+00  0.000000E+00  0.000000E+00  0.000000E+00  0.000000E+00  0.000000E+00  0.000000E+00  0.000000E+00  0.000000E+00  0.000000E+00  0.000000E+00       Funtag_D
      88  3.911204E+05  3.502986E+05       40821.8       5.60066     0.0768659       1.90308      0.182561      0.240843    0.00272962   -0.00137718    0.00828391       1.04489       Opttag_D
      88       82.6584      0.947289       1.00000  0.000000E+00       1.00000  0.000000E+00      0.947413       99.0488       1.00000       33.0523  0.000000E+00      -1.31759  0.000000E+00      -1.31759  0.000000E+00  0.000000E+00       2.63518       83.1364     -0.105908        1       Scatag_D
      88       1935.05       5962.11  0.000000E+00  0.000000E+00       2703.49  0.000000E+00       2382.92       8239.43  0.000000E+00       13038.0       51.6484       6509.16 -1.000000E+10 -1.000000E+10 -1.000000E+10 -1.000000E+10 -1.000000E+10  0.000000E+00  0.000000E+00  0.000000E+00  0.000000E+00  0.000000E+00  0.000000E+00  0.000000E+00  0.000000E+00  0.000000E+00  0.000000E+00  0.000000E+00  0.000000E+00       Funtag_D
      89  3.911134E+05  3.502892E+05       40824.2       5.37747     0.0770649       1.90197      0.182555      0.240849    0.00275604   -0.00140182    0.00828301       1.04491       Opttag_D
      89       82.6598      0.947291       1.00000  0.000000E+00       1.00000  0.000000E+00      0.947398       99.0733       1.00000       33.0508  0.000000E+00      -1.31804  0.000000E+00      -1.31804  0.000000E+00  0.000000E+00       2.63609       83.1364     -0.105908        1       Scatag_D
      89       1934.63       5962.35  0.000000E+00  0.000000E+00       2703.71  0.000000E+00       2383.15       8240.32  0.000000E+00       13038.7       51.6768       6509.69 -1.000000E+10 -1.000000E+10 -1.000000E+10 -1.000000E+10 -1.000000E+10  0.000000E+00  0.000000E+00  0.000000E+00  0.000000E+00  0.000000E+00  0.000000E+00  0.000000E+00  0.000000E+00  0.000000E+00  0.000000E+00  0.000000E+00  0.000000E+00       Funtag_D
      90  3.911059E+05  3.502790E+05       40826.9       4.92456     0.0772457       1.89912      0.182550      0.240856    0.00278491   -0.00142077    0.00828196       1.04494       Opttag_D
      90       82.6610      0.947239       1.00000  0.000000E+00       1.00000  0.000000E+00      0.947384       99.0961       1.00000       33.0492  0.000000E+00      -1.31825  0.000000E+00      -1.31825  0.000000E+00  0.000000E+00       2.63649       83.1364     -0.105908        1       Scatag_D
      90       1934.15       5962.66  0.000000E+00  0.000000E+00       2703.86  0.000000E+00       2383.40       8241.44  0.000000E+00       13039.6       51.6863       6510.16 -1.000000E+10 -1.000000E+10 -1.000000E+10 -1.000000E+10 -1.000000E+10  0.000000E+00  0.000000E+00  0.000000E+00  0.000000E+00  0.000000E+00  0.000000E+00  0.000000E+00  0.000000E+00  0.000000E+00  0.000000E+00  0.000000E+00  0.000000E+00       Funtag_D
      91  3.910967E+05  3.502594E+05       40837.3       6.29465     0.0775598       1.89182      0.182538      0.240871    0.00283999   -0.00145857    0.00828193       1.04502       Opttag_D
      91       82.6629      0.947071       1.00000  0.000000E+00       1.00000  0.000000E+00      0.947360       99.1346       1.00000       33.0458  0.000000E+00      -1.31825  0.000000E+00      -1.31825  0.000000E+00  0.000000E+00       2.63651       83.1364     -0.105908        1       Scatag_D
      91       1934.13       5963.62  0.000000E+00  0.000000E+00       2704.05  0.000000E+00       2383.83       8246.83  0.000000E+00       13042.0       51.6445       6511.26 -1.000000E+10 -1.000000E+10 -1.000000E+10 -1.000000E+10 -1.000000E+10  0.000000E+00  0.000000E+00  0.000000E+00  0.000000E+00  0.000000E+00  0.000000E+00  0.000000E+00  0.000000E+00  0.000000E+00  0.000000E+00  0.000000E+00  0.000000E+00       Funtag_D
      92  3.910885E+05  3.502425E+05       40846.0       6.12890     0.0779081       1.88753      0.182527      0.240886    0.00288746   -0.00148573    0.00828339       1.04512       Opttag_D
      92       82.6646      0.946959       1.00000  0.000000E+00       1.00000  0.000000E+00      0.947337       99.1714       1.00000       33.0428  0.000000E+00      -1.31838  0.000000E+00      -1.31838  0.000000E+00  0.000000E+00       2.63676       83.1364     -0.105908        1       Scatag_D
      92       1934.81       5964.79  0.000000E+00  0.000000E+00       2704.24  0.000000E+00       2384.16       8249.78  0.000000E+00       13044.2       51.5854       6512.36 -1.000000E+10 -1.000000E+10 -1.000000E+10 -1.000000E+10 -1.000000E+10  0.000000E+00  0.000000E+00  0.000000E+00  0.000000E+00  0.000000E+00  0.000000E+00  0.000000E+00  0.000000E+00  0.000000E+00  0.000000E+00  0.000000E+00  0.000000E+00       Funtag_D
      93  3.910812E+05  3.502309E+05       40850.3       5.13734     0.0781551       1.88489      0.182519      0.240898    0.00292008   -0.00150795    0.00828451       1.04522       Opttag_D
      93       82.6655      0.946863       1.00000  0.000000E+00       1.00000  0.000000E+00      0.947321       99.1953       1.00000       33.0407  0.000000E+00      -1.31840  0.000000E+00      -1.31840  0.000000E+00  0.000000E+00       2.63680       83.1364     -0.105908        1       Scatag_D
      93       1935.33       5965.91  0.000000E+00  0.000000E+00       2704.35  0.000000E+00       2384.08       8250.58  0.000000E+00       13045.8       51.5335       6512.68 -1.000000E+10 -1.000000E+10 -1.000000E+10 -1.000000E+10 -1.000000E+10  0.000000E+00  0.000000E+00  0.000000E+00  0.000000E+00  0.000000E+00  0.000000E+00  0.000000E+00  0.000000E+00  0.000000E+00  0.000000E+00  0.000000E+00  0.000000E+00       Funtag_D
      94  3.910725E+05  3.502104E+05       40862.0       5.57239     0.0786699       1.88101      0.182507      0.240921    0.00297761   -0.00155721    0.00828647       1.04542       Opttag_D
      94       82.6674      0.946749       1.00000  0.000000E+00       1.00000  0.000000E+00      0.947289       99.2428       1.00000       33.0369  0.000000E+00      -1.31865  0.000000E+00      -1.31865  0.000000E+00  0.000000E+00       2.63731       83.1364     -0.105908        1       Scatag_D
      94       1936.25       5968.17  0.000000E+00  0.000000E+00       2704.58  0.000000E+00       2384.06       8255.11  0.000000E+00       13049.0       51.4914       6513.37 -1.000000E+10 -1.000000E+10 -1.000000E+10 -1.000000E+10 -1.000000E+10  0.000000E+00  0.000000E+00  0.000000E+00  0.000000E+00  0.000000E+00  0.000000E+00  0.000000E+00  0.000000E+00  0.000000E+00  0.000000E+00  0.000000E+00  0.000000E+00       Funtag_D
      95  3.910671E+05  3.501910E+05       40876.0       7.64826     0.0792481       1.87708      0.182495      0.240945    0.00303219   -0.00162157    0.00828664       1.04562       Opttag_D
      95       82.6693      0.946688       1.00000  0.000000E+00       1.00000  0.000000E+00      0.947255       99.2939       1.00000       33.0334  0.000000E+00      -1.31893  0.000000E+00      -1.31893  0.000000E+00  0.000000E+00       2.63785       83.1364     -0.105908        1       Scatag_D
      95       1936.33       5970.49  0.000000E+00  0.000000E+00       2704.88  0.000000E+00       2384.44       8261.42  0.000000E+00       13052.5       51.5250       6514.45 -1.000000E+10 -1.000000E+10 -1.000000E+10 -1.000000E+10 -1.000000E+10  0.000000E+00  0.000000E+00  0.000000E+00  0.000000E+00  0.000000E+00  0.000000E+00  0.000000E+00  0.000000E+00  0.000000E+00  0.000000E+00  0.000000E+00  0.000000E+00       Funtag_D
      96  3.910604E+05  3.501874E+05       40873.0       5.38184     0.0793711       1.87699      0.182494      0.240951    0.00304255   -0.00163775    0.00828516       1.04563       Opttag_D
      96       82.6696      0.946684       1.00000  0.000000E+00       1.00000  0.000000E+00      0.947248       99.3043       1.00000       33.0325  0.000000E+00      -1.31907  0.000000E+00      -1.31907  0.000000E+00  0.000000E+00       2.63814       83.1364     -0.105908        1       Scatag_D
      96       1935.64       5970.62  0.000000E+00  0.000000E+00       2704.94  0.000000E+00       2384.44       8258.61  0.000000E+00       13053.0       51.5629       6514.25 -1.000000E+10 -1.000000E+10 -1.000000E+10 -1.000000E+10 -1.000000E+10  0.000000E+00  0.000000E+00  0.000000E+00  0.000000E+00  0.000000E+00  0.000000E+00  0.000000E+00  0.000000E+00  0.000000E+00  0.000000E+00  0.000000E+00  0.000000E+00       Funtag_D
      97  3.910516E+05  3.501747E+05       40876.9       4.83956     0.0797981       1.87601      0.182485      0.240968    0.00307833   -0.00169522    0.00828330       1.04575       Opttag_D
      97       82.6706      0.946669       1.00000  0.000000E+00       1.00000  0.000000E+00      0.947222       99.3404       1.00000       33.0299  0.000000E+00      -1.31941  0.000000E+00      -1.31941  0.000000E+00  0.000000E+00       2.63883       83.1364     -0.105908        1       Scatag_D
      97       1934.77       5971.98  0.000000E+00  0.000000E+00       2705.17  0.000000E+00       2384.63       8259.18  0.000000E+00       13055.0       51.6565       6514.54 -1.000000E+10 -1.000000E+10 -1.000000E+10 -1.000000E+10 -1.000000E+10  0.000000E+00  0.000000E+00  0.000000E+00  0.000000E+00  0.000000E+00  0.000000E+00  0.000000E+00  0.000000E+00  0.000000E+00  0.000000E+00  0.000000E+00  0.000000E+00       Funtag_D
      98  3.910451E+05  3.501601E+05       40885.0       5.28546     0.0802596       1.87485      0.182476      0.240985    0.00311939   -0.00175788    0.00828399       1.04589       Opttag_D
      98       82.6717      0.946611       1.00000  0.000000E+00       1.00000  0.000000E+00      0.947197       99.3779       1.00000       33.0269  0.000000E+00      -1.31955  0.000000E+00      -1.31955  0.000000E+00  0.000000E+00       2.63910       83.1364     -0.105908        1       Scatag_D
      98       1935.09       5973.57  0.000000E+00  0.000000E+00       2705.43  0.000000E+00       2384.81       8261.94  0.000000E+00       13057.3       51.6826       6515.19 -1.000000E+10 -1.000000E+10 -1.000000E+10 -1.000000E+10 -1.000000E+10  0.000000E+00  0.000000E+00  0.000000E+00  0.000000E+00  0.000000E+00  0.000000E+00  0.000000E+00  0.000000E+00  0.000000E+00  0.000000E+00  0.000000E+00  0.000000E+00       Funtag_D
      99  3.910345E+05  3.501225E+05       40911.9       7.57018     0.0815171       1.87355      0.182454      0.241028    0.00322499   -0.00195373    0.00828770       1.04633       Opttag_D
      99       82.6741      0.946465       1.00000  0.000000E+00       1.00000  0.000000E+00      0.947131       99.4743       1.00000       33.0192  0.000000E+00      -1.31973  0.000000E+00      -1.31973  0.000000E+00  0.000000E+00       2.63945       83.1364     -0.105908        1       Scatag_D
      99       1936.83       5978.64  0.000000E+00  0.000000E+00       2706.14  0.000000E+00       2385.29       8271.82  0.000000E+00       13063.5       51.7101       6518.03 -1.000000E+10 -1.000000E+10 -1.000000E+10 -1.000000E+10 -1.000000E+10  0.000000E+00  0.000000E+00  0.000000E+00  0.000000E+00  0.000000E+00  0.000000E+00  0.000000E+00  0.000000E+00  0.000000E+00  0.000000E+00  0.000000E+00  0.000000E+00       Funtag_D

   MaxLik Scaling Cycle 0106

   Wilson plot

   Statistics in resolution bins after refinement cycle 0003
```


|  |  |
| --- | --- |
|  | - Rxpct factors plot- Rxpct factors table- <Log-Lik Gain>- Ln Sigma A- Correlation Coefficients |


```
   Iters     Tot_Funct    Xray_Funct    Geom_Funct          Grms     rmsD2init     maxD2init             R         Rfree           LLG       LLGfree     rms(bond)    rms(angle)
   Iters             K   B_IMPF_FRAG        K_MISS   B_TEMP_MISS   K_IMPF_MISS   B_IMPF_MISS        K_SOLV   B_TEMP_SOLV   K_IMPF_SOLV   B_IMPF_SOLV        B_TEMP     B_TEMP_11     B_TEMP_12     B_TEMP_22     B_TEMP_13     B_TEMP_23     B_TEMP_33      K_WILSON      B_WILSON      FGCYCLES
   Iters          BOND         ANGLE      IMPROPER       TORSION    SINTORSION      TRIGONAL        PLANAR  BCORRELATION       CONTACT     IDEALDIST        CHIRAL    SIMILARITY       OCCSSUM  UTILDISTANCE     UTILANGLE   UTILTORSION        RHOFIT        ADP-01        ADP-02        ADP-03        ADP-04        ADP-05        ADP-06        ADP-07        ADP-08        ADP-09        ADP-10      RHOFITPC            QM
     100  3.910250E+05  3.501051E+05       40919.9       6.61307     0.0821095       1.87412      0.182443      0.241045    0.00327398   -0.00205056    0.00829149       1.04642       Opttag_D
     100       82.6748      0.946380       1.00000  0.000000E+00       1.00000  0.000000E+00      0.947105       99.5146       1.00000       33.0145  0.000000E+00      -1.31962  0.000000E+00      -1.31962  0.000000E+00  0.000000E+00       2.63925       83.1364     -0.105908        1       Scatag_D
     100       1938.60       5979.64  0.000000E+00  0.000000E+00       2706.47  0.000000E+00       2385.55       8273.01  0.000000E+00       13065.9       51.6679       6519.10 -1.000000E+10 -1.000000E+10 -1.000000E+10 -1.000000E+10 -1.000000E+10  0.000000E+00  0.000000E+00  0.000000E+00  0.000000E+00  0.000000E+00  0.000000E+00  0.000000E+00  0.000000E+00  0.000000E+00  0.000000E+00  0.000000E+00  0.000000E+00       Funtag_D
   Geometry Restraint Deviations SCREEN Information

 
 No -bcorrel_zeroocchydrogens_factor flag found - keeping default: will set B for OCC=0 hydrogens to    1.200 times heavy-atom B


 TLS information. Total number of groups:     2


 Group   Natoms    FixRB   FixTLS   Group Tag    Group Spec
     1     2783      Y        Y     tls1    { A|* }
     2     2770      Y        Y     tls2    { B|* }
 
NOTE BUSTER_TLS_SET tls1 { A|* }
NOTE BUSTER_TLS_T   tls1     -2.44720E-01 -1.33885E-01  3.49464E-01 -7.26320E-02  1.36549E-02 -3.31073E-02
NOTE BUSTER_TLS_L   tls1      2.21033E+00  2.43686E+00  1.90206E+00 -4.70482E-01 -1.42181E+00  6.26775E-01
NOTE BUSTER_TLS_S   tls1      1.26526E-01 -3.75851E-01  4.70780E-01 -3.29027E-01  1.72095E-01 -3.71904E-01  6.80978E-02 -3.47299E-01
NOTE BUSTER_TLS_O   tls1     -2.31731E+01 -8.40215E+01  1.10770E+01
NOTE BUSTER_TLS_FIX tls1 ALL
 
NOTE BUSTER_TLS_SET tls2 { B|* }
NOTE BUSTER_TLS_T   tls2     -3.51018E-01 -2.65873E-01  4.05020E-01  2.48330E-02  3.11379E-02 -4.04722E-02
NOTE BUSTER_TLS_L   tls2      1.80307E+00  1.69393E+00  1.39140E+00  8.83117E-01 -1.11732E+00 -9.56109E-01
NOTE BUSTER_TLS_S   tls2     -1.42162E-02  9.70000E-02 -1.62700E-01  8.39808E-02  2.10687E-01  5.67053E-02 -9.55320E-02  7.37692E-02
NOTE BUSTER_TLS_O   tls2      2.22040E+01 -7.22942E+01  3.58164E+00
NOTE BUSTER_TLS_FIX tls2 ALL

 For final position, Geometry function summary
     term                           number     weight       rms        GooF      function     fn/numb
     ================================================================================================
     BOND bond lengths (angs)       5668       2.000      0.0083       0.414      1938.60       0.342
     ANGL bond angles (degs)        7691       2.000        1.05       0.623      5979.64       0.777
     TORS split-harmonic tors       1275       0.000       18.94       1.191         0.00       0.000
     SINTOR sinusoidal tors         1275       2.000       18.94                  2706.47       2.123
     PLAN general plane (angs)       967       5.000      0.0140       0.702      2385.55       2.467
     BCORrelations (angs^2)         5668      10.000       1.070       0.382      8273.01       1.460
     CONT (bad contacts)               0       5.000                                 0.00
     IDEAL(ideal-dist contact)      5667       4.000                             13065.86
     CHIRAL (gelly semiharmon)       740       5.000                                51.67
     SIM similarity restraint for NCS and/or TARGET                               6519.10
     OCCSUM restraint on sum of occupanies                                           0.00       as none defined
     DISTAN utility restraints interatomic distance                                  0.00       as none defined
     UTILANGLE utility angle restraints                                              0.00       as none defined
     UTILTOR utility restraints on torsion angles                                    0.00       as none defined
     Peptide omega torsion angles                           2.87 degs - monitor only

 Weighted rms      0.0083         1.046  (temporary rms bond and angle for BUSTER to pick up!)
 Weighted intra-residual rms(bond) for StdProtein (excluding H)      0.0081
 Weighted intra-residual rms(bond) for Buffer     (excluding H)      0.0030
 Outputing geometry progress graph in plotmtv format to file:Geom_Res.mtv

 ############### Running final geometry/screen analysis (screen_final.txt)

 s/r gelly normal termination
```

Statistics and scale factors vs. cycle number
```

|  |  |  |
| --- | --- | --- |
|  | - Rxpct factors- <Log-Lik Gain>- GEOM Residuals- Ln Sigma_A_ | - Scale Factors- B Factors- Imperfection Factors |

```
 Writing F_calc(model+solvent) to complete.mtz

   Final Geometry Information


---


### 2. Final results

Statistics and scale factors vs. cycle number
```

|  |  |  |
| --- | --- | --- |
|  | - Rxpct factors- <Log-Lik Gain>- GEOM Residuals- Ln Sigma_A_ | - Scale Factors- B Factors- Imperfection Factors |

```
   Average Figure of Merit
   Structure Factor Amplitudes


 # normal termination

  Program stopped.
```

closesok message: time spent in tree = 1709.91 (user) 33.64 (sys)

---

# Normal termination

```
 Resources usage summary:
     Time spent in client ...................  1709.91 (user)       33.67 (system)
     Time spent in scripts (    27 calls) ...     2.31 (user)        0.16 (system)
     Time spent in server ...................     0.00 (user)        0.01 (system)

     Max resident memory used (kb) .......... 381720
     Number of page faults .................. 0 (major)    1007990 (minor)
     Number of context switches ............. 12172 (niced)    6681 (other)
```
